# Site-specific modification and segmental isotope labelling of HMGN1 reveals long-range conformational perturbations caused by posttranslational modifications†

**DOI:** 10.1039/d0cb00175a

**Published:** 2021-01-05

**Authors:** Gerhard Niederacher, Debra Urwin, Yasmin Dijkwel, David J. Tremethick, K. Johan Rosengren, Christian F. W. Becker, Anne C. Conibear

**Affiliations:** Faculty of Chemistry, Institute of Biological Chemistry, University of Vienna Währinger Straße 38 1090 Vienna Austria; John Curtin School of Medical Research, Department of Genome Sciences, The Australian National University ACT 2601 Australia; School of Biomedical Sciences, The University of Queensland Brisbane QLD 4072 Australia a.conibear@uq.edu.au +61-7-3365-1738

## Abstract

Interactions between histones, which package DNA in eukaryotes, and nuclear proteins such as the high mobility group nucleosome-binding protein HMGN1 are important for regulating access to DNA. HMGN1 is a highly charged and intrinsically disordered protein (IDP) that is modified at several sites by posttranslational modifications (PTMs) – acetylation, phosphorylation and ADP-ribosylation. These PTMs are thought to affect cellular localisation of HMGN1 and its ability to bind nucleosomes; however, little is known about how these PTMs regulate the structure and function of HMGN1 at a molecular level. Here, we combine the chemical biology tools of protein semi-synthesis and site-specific modification to generate a series of unique HMGN1 variants bearing precise PTMs at their N- or C-termini with segmental isotope labelling for NMR spectroscopy. With access to these precisely-defined variants, we show that PTMs in both the N- and C-termini cause changes in the chemical shifts and conformational populations in regions distant from the PTM sites; up to 50–60 residues upstream of the PTM site. The PTMs investigated had only minor effects on binding of HMGN1 to nucleosome core particles, suggesting that they have other regulatory roles. This study demonstrates the power of combining protein semi-synthesis for introduction of site-specific PTMs with segmental isotope labelling for structural biology, allowing us to understand the role of PTMs with atomic precision, from both structural and functional perspectives.

## Abstract 2

Using protein semi-synthesis, segmentally isotope-labelled variants of nucleosome-binding protein HMGN1 were generated with site-specific posttranslational modifications to explore their structural and functional effects.

## Graphical abstract

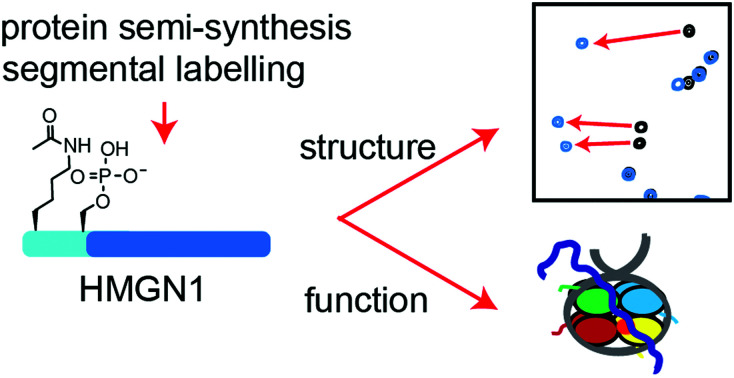

## Introduction

**Fig. 1:**
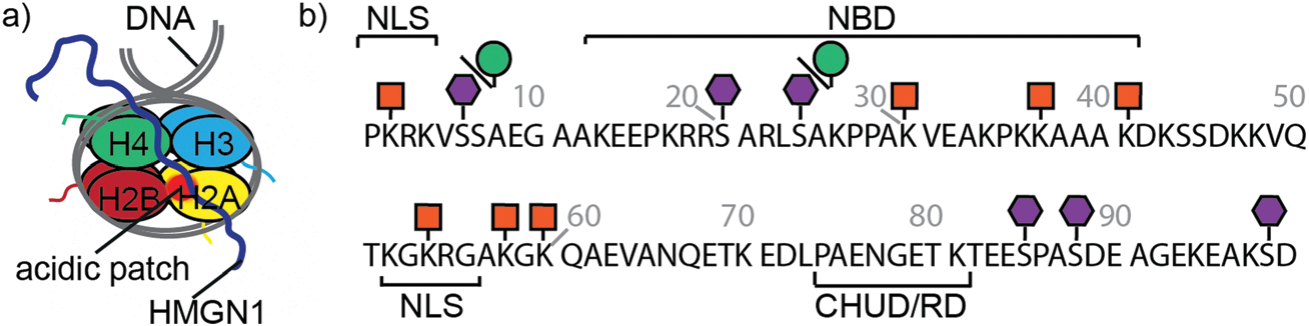
HMGN1 interacts with nucleosomes and bears phosphorylation, ADP ribosylation and acetylation PTMs. (a) Schematic structure of a nucleosome comprising dimers of each of the four histones H2A, H2B, H3 and H4 wrapped by DNA. HMGN1 (dark blue line) is an intrinsically disordered protein that binds to nucleosomes. Orientation and interaction of HMGN1 is based on that reported for HMGN2.^26^ (b) Protein sequence, domains and PTMs of HMGN1.^27,28^ Amino acids are represented by their one-letter codes and residue numbers are shown in grey. NLS = nuclear localization signal, NBD = nucleosome-binding domain, CHUD/RD = chromatin unfolding domain/regulatory domain. Lysine acetylation is represented by an orange square, serine phosphorylation by a purple hexagon and ADP ribosylation by a green circle.

Eukaryotic cells package DNA into chromatin, a dynamic and complex structure made up of nucleosomes and non-histone proteins.^1–3^ Nucleosome cores comprise ∼147 base pairs of DNA wrapped around an octamer of two copies of each of the four histones H2A, H2B, H3 and H4 (Fig. 1a).^4,5^ Linker DNA joins nucleosome cores into arrays and interacts with linker histone H1, forming higher order chromatin structures.^4^ Structural biology techniques, as well as single-molecule and biophysical methods, have given insights into the structural dynamics of nucleosomes, showing that chromatin switches between multiple states, each with specific biological functions.^6,7^ Associated with these chromatin structures are non-histone proteins, such as the high-mobility group proteins, which modulate chromatin architecture and dynamics, thereby influencing a range of biological processes.^8,9^

High mobility group (HMG) proteins are part of the dynamic network of architectural proteins in the nucleus. They are characterised by a high content of charged residues and are divided into three families – HMGA, HMGB and HMGN – based on their DNA-binding domains.^9,10^ The nucleosome-binding HMGN family comprises five proteins (HMGN1, 2, 3, 4 and 5), all of which have a nuclear localisation signal (NLS) split into two segments, a conserved nucleosome-binding domain (NBD) and a negatively-charged C-terminal chromatin regulatory domain (CHUD/RD, Fig. 1b).^11^ HMGN1 and HMGN2 are ubiquitously expressed and are thought to regulate transcription by modulating chromatin remodelling, having an important role in DNA repair, regulation of histone posttranslational modifications (PTMs) and maintenance of cell identity.^12–16^ Although the precise mechanisms by which HMGN proteins modulate epigenetic processes are still not clear, several studies indicate that these nucleosomal proteins regulate cell-type-specific gene expression, and therefore have potential implications in developmental processes and disease.^11^ For example, HMGN proteins have been reported to function as alarmins,^17–19^ to have both pro-^20^ and anti-tumour^21^ activities and to have roles in DNA repair,^22,23^ immune regulation^17^ and Down syndrome.^11,24^ These studies, amongst others, highlight the need to understand the molecular-level interactions of HMGN1.

HMGN proteins are intrinsically disordered and interact dynamically with chromatin.^13–15^ Circular dichroism studies indicated that HMGN1 and HMGN2 binding caused changes in nucleosome structure, but that these structural changes differed for the two variants.^25^ A detailed study of the interaction between HMGN2 and nucleosomes was carried out using methyl-TROSY NMR and showed that HMGN2 interacts with the acidic patch (comprising six acidic amino acids of H2A and two acidic amino acids of H2B located on the nucleosome surface) *via* two conserved arginine residues (Arg22 and Arg26) in the NBD as well as with the nucleosomal DNA *via* its lysine-rich C-terminal region (Fig. 1a).^26^ Based on modelling studies, the authors proposed that phosphorylation of Ser24 and Ser28 of HMGN2 during mitosis causes electrostatic repulsion with negatively charged residues in the acidic patch, leading to dissociation of the complex.^26^

Posttranslational modifications (PTMs) of HMGN proteins, predominantly serine phosphorylation and lysine acetylation, have been detected at several sites, as shown for HMGN1 in Fig. 1b.^27–29^ It is proposed that these PTMs regulate the nucleosome binding and subcellular location of HMGN proteins and thereby the accessibility of chromatin and modification of histones.^30–32^ However, the PTMs and enzymes responsible appear to differ between the two most widely-studied variants, HMGN1 and HMGN2.^28,33^ The enzymes that install PTMs on HMGN1 also modify histones. Therefore, access of these enzymes and other histone modifiers might be modulated by HMGN1 binding to the nucleosome.^29,32^ More recently, in a broad search of ADP ribosylation sites, which demonstrated that this PTM is widespread on histones, serine ADP-ribosylation was also reported for HMGN1 at residues Ser6 and Ser24.^34^ Although proteomics approaches have identified PTM sites on HMGN proteins, many questions remain about effects of these PTMs, individually and in combination. Access to site-specifically modified HMGN variants will therefore be important to understand how these PTMs and combinations of PTMs influence HMGN interactions with nucleosomes and modulate chromatin dynamics. The value of access to site-specifically modified variants for elucidating chromatin dynamics and transcriptional regulation has been demonstrated in numerous studies on histones,^35–37^ however such studies have not yet been widely carried out on non-histone proteins that are associated with chromatin nor considered structural effects of their PTMs.

The chemical biology tools of protein semi-synthesis and site-specific modification provide unique access to homogeneous proteins bearing PTMs.^38^ Such proteins are difficult to obtain from natural or recombinant sources and are crucial for understanding the structural and functional roles of PTMs. Typically, a segment of the protein is synthesized using solid phase peptide synthesis (SPPS), which allows for introduction of the desired PTM(s) in precise locations. The remaining segment(s) of the protein are produced recombinantly and ligated to the synthetic segment *via* native chemical ligation, or a derivative thereof.^39,40^ Recent developments and combinations with enzymatic ligations have greatly expanded the scope of protein synthesis and semi-synthesis and allow for incorporation of site-specific PTMs and combinations of PTMs, in principle, in any part of the protein.^38,39,41^ Furthermore, protein semi-synthesis also allows for introduction of site-specific and segmental isotope labels that are often essential for studying structural and binding properties of proteins by NMR spectroscopy.^42^ NMR is a powerful technique for studying dynamic and intrinsically disordered proteins like HMGN1, which are not amenable to X-ray crystallography or cryo-electron microscopy.^26,43^ The potential of combining site-specific protein modification and segmental isotope labelling for NMR spectroscopy has not yet been widely explored but has the opportunity to provide a unique perspective on how PTMs cause structural or conformational changes to proteins and to link these to biological consequences.

Here we combine site-specific modification and segmental isotope labelling to provide unique access to HMGN1 variants, allowing us to explore the structural and functional effects of PTMs on this intrinsically disordered protein. Access to modified and unmodified HMGN1 with various isotope labelling patterns allows for the first atomic-resolution information on PTMs and combinations of PTMs, and comparison of their activities. We report the NMR chemical shift assignments of HMGN1, facilitated by segmental labelling and show how the labelled variants allow conformational perturbations to be observed. PTMs in the N- and C-terminal segments give rise to shifts in signals from domains that are distant in terms of primary sequence, some of which form part of the nucleosome binding domain. The site-specifically modified HMGN1 variants were also compared in functional assays for their nucleosome core-binding abilities. Similar binding of the HMGN1 variants to nucleosome core particles, suggests that the PTMs might have more subtle effects or regulate interactions with other partners, for example linker DNA, or act as a code for other nucleosomal binding proteins.

## Materials and methods

### Solid phase peptide synthesis

Solid phase peptide synthesis (SPPS) of HMGN1 segments was carried out using 9-fluorenylmethoxycarbonyl (Fmoc) chemistry, either manually or on an automated synthesizer. For manual syntheses and automated syntheses on a PTI Tribute synthesizer, Fmoc-protected building blocks were coupled using 2-(1*H*-benzotriazol-1-yl)-1,1,3,3-tetramethyluronium hexafluorophosphate (HBTU, 2.4 equiv., 0.5 M in DMF) as activator in combination with diisopropylethylamine (DIPEA, 5 equiv.) as base at room temperature for 20–30 min. N-terminal deprotection was achieved with piperidine [20% in dimethylformamide (DMF), 2 × 5 min]. Peptides synthesized on a Liberty Blue microwave peptide synthesizer (CEM) were coupled with diisopropyl carbodiimide (DIC, 0.25 M in DMF) and OxymaPure (0.5 M in DMF), using 5 equiv. of amino acid and microwave irradiation for 4 min at 90 °C. Deprotection was carried out using piperazine (25% in DMF). C-terminally amidated peptides were synthesized on Rink amide resin and C-terminal acid peptides were synthesized on pre-loaded Wang or Tentagel resins. Short random-coil peptides were N-terminally acetylated with acetic anhydride/DCM/DIPEA 10 : 85 : 5 (2 × 5 min). Peptides with C-terminal hydrazides were synthesized on hydrazide-linked resin using the method of Zheng *et al.*^44^ Modified residues were installed using Fmoc-Ser(PO(OBzl)OH)-OH (CEM) or Fmoc-Lys(ac)-OH (Merck) and were coupled without microwave irradiation. After drying, peptides were cleaved from the resin with trifluoroacetic acid (TFA)/dimethylsulfide (DMS)/triisopropylsilane (TIPS)/H_2_O 90 : 5 : 2.5 : 2.5 for 2–3 h and then precipitated with diethyl ether and pelleted by centrifugation. Crude peptides were dissolved in acetonitrile (ACN)/water 1 : 1, lyophilized and then purified by RP-HPLC on either C4 or C18 columns using a gradient of ACN in water with 0.1% TFA. Purity was analysed by electrospray mass spectrometry in positive ion mode and by analytical RP-HPLC.

### Construct design and expression of recombinant protein segments

#### Recombinant expression and purification of full-length HMGN1

A DNA construct comprising a hexa-histidine-tag followed by a tobacco etch virus (TEV) cleavage site (ENLYFQ|S) and full-length HMGN1 (His_6_-TEV-HMGN1, Eurofins Genomics, full sequence in Supplementary Data S1, ESI†) was cloned into a pET21a plasmid *via* NdeI and XhoI restriction sites. Expression was carried out in *E. coli* BL21(DE3) using 2YT medium (16 g L^−1^ tryptone, 10 g L^−1^ yeast extract, 5 g L^−1^ NaCl) containing 100 μg mL^−1^ ampicillin. Overnight cultures were diluted to OD_600_ ∼ 0.2, grown at 37 °C until OD_600_ ∼ 0.7 and protein overexpression was induced with 1 mM isopropyl thiogalactopyranoside (IPTG). After 4 h, cells were harvested and cell pellets resuspended in TBS (50 mM Tris, 150 mM NaCl, pH 7.5) buffer and lysed by passing twice through a high-pressure cell disruptor (Constant Systems). The lysate was centrifuged and the supernatant loaded on a NiNTA column (GE HisTrap HP 5 mL) equilibrated with TBS. Protein was eluted from the column with a gradient of 0–400 mM imidazole in TBS over 60 min. Fractions containing His_6_-TEV-HMGN1 were identified by SDS-PAGE, pooled and dialyzed against TBS. The His_6_-tag was removed by TEV protease cleavage at 4 °C overnight with a 1 : 20 v/v ratio of TEV protease and 1 mM dithiothreitol (DTT). Finally, full length HMGN1 was purified by RP-HPLC on a C4 column with a gradient of 5–40% ACN in water with 0.1% TFA over 35 min at a flow rate of 3 mL min^−1^.

#### Recombinant expression and purification of HMGN1(1–65)-thioester

A synthetic HMGN1(1–65) DNA sequence (Eurofins Genomics, full sequence in Supplementary Data S1, ESI†) was inserted upstream of the *Mycobacterium xenopi* DNA gyrase A (Mxe GyrA) intein, His_7_-tag and a chitin-binding domain (CBD) in a pTXB1 (New England Biolabs) vector *via* NdeI and SpeI restriction sites. Recombinant protein expression was carried out in *E. coli* BL21(DE3) Rosetta2 (Novagen) cells in 2YT medium (16 g L^−1^ tryptone, 10 g L^−1^ yeast extract, 5 g L^−1^ NaCl) containing 100 μg mL^−1^ ampicillin and 30 μg mL^−1^ chloramphenicol. Overnight cultures were diluted to OD ∼ 0.2, grown at 37 °C until OD ∼ 0.7 and protein overexpression was induced with 1 mM IPTG. After 4 h, cells were harvested by centrifugation and cell pellets were resuspended in TBS buffer and lysed by passing twice through a high-pressure cell disruptor (Constant Systems). Solubilization of inclusion bodies was achieved by treatment with 8 M guanidinium hydrochloride (Gdn·HCl) overnight at room temperature followed by NiNTA affinity purification on an Äkta Prime Plus FPLC system using a GE HisTrap HP 5 mL column equilibrated with 6 M Gdn·HCl/TBS buffer. Bound protein was eluted from the column with a gradient from 0–500 mM imidazole over 1 h at a flow rate of 1 mL min^−1^. The fractions containing the HMGN1(1–65)-Mxe-His_7_-CBD fusion protein were identified by SDS-PAGE, pooled and concentrated with centrifugal filters (Merck Amicon, 10 kDa MWCO). Buffer exchange to 8 M urea was performed using Sephadex G-25 PD10 columns (GE Healthcare). The urea concentration of the obtained sample was reduced from 8 M to 4 M by dilution with TBS. Mxe-intein cleavage was induced by an additional 1 : 1 dilution with 1 M mercaptoethane sulfonate sodium salt (MesNa) in TBS (final concentration: 500 mM MesNa, 2 M urea in TBS) overnight. HMGN1(1–65)-thioester was purified by RP-HPLC on a C4 column (Kromasil) with a gradient of 5–65% ACN in water with 0.1% TFA over 30 min at a flow rate of 3 mL min^−1^.

#### Recombinant expression and purification of HMGN1(11–99)_A11C

A pET21a plasmid containing the His_6_-TEV-HMGN1(11–99)_A11C sequence (BioCat, full sequence in Supplementary Data S1, ESI†) was transformed into *E. coli* BL21(DE3) Rosetta2 (Novagen). His_6_-TEV represents a hexa-histidine-Tag followed by the modified recognition sequence for TEV protease (ENLYFQ|C) to obtain an N-terminal cysteine after TEV protease cleavage. Protein expression was performed in 2YT medium (16 g L^−1^ tryptone, 10 g L^−1^ yeast extract, 5 g L^−1^ NaCl) containing 100 μg mL^−1^ ampicillin and 30 μg mL^−1^ chloramphenicol. Overnight cultures were diluted to OD ∼ 0.2, grown at 37 °C until OD ∼ 0.7 and protein overexpression was induced with 1 mM IPTG. After 4 h, cells were harvested, and cell pellets were resuspended in TBS buffer and lysed by passing twice through a high-pressure cell disruptor (Constant Systems). The lysate was centrifuged and the supernatant was loaded on a NiNTA column (GE HisTrap HP 5 mL), equilibrated with 10 mM imidazole in TBS. Protein was eluted from the column with a gradient from 10–500 mM imidazole in TBS over 1 h. Fractions containing His_6_-TEV-HMGN1(11–99) were identified by SDS-PAGE, pooled and dialyzed against TBS. For the removal of the purification tag and the release of the N-terminal cysteine, 1 mM tris-carboxylethyl phosphine (TCEP) and TEV protease (1 : 20 ratio) were added and the protein was cleaved at 4 °C overnight. HMGN1(11–99) was purified by RP-HPLC on a C4 column (Kromasil) with a gradient of 5–45% gradient of ACN in water with 0.1% TFA in 60 min at a flow rate of 3 mL min^−1^.

#### Recombinant expression of ^15^N- and ^13^C/^15^N-labeled proteins

For expression of ^15^N-labeled proteins, cultures were grown to OD 0.7 in 2YT, pelleted by centrifugation and the cell pellet was washed with 1 L M9 buffer (3 g L^−1^ KH_2_PO_4_, 12.8 g L^−1^ Na_2_HPO_4_ × 7H_2_O, 0.5 g L^−1^ NaCl) and resuspended in a quarter of the initial volume of minimal medium containing 4 g L^−1^ glucose [unlabeled or uniformly ^13^C-labeled (U–^13^C-6, 99%, Cambridge Isotope Laboratories CLM-1396-10)], 1 g L^−1 15^NH_4_Cl (^15^N, 99%, Cambridge Isotope Laboratories NLM-467-25), 10 mL L^−1^ Basal Medium Eagle (Sigma), 2 mM MgSO_4_, and 0.1 mM CaCl_2_ in M9 buffer. After a 1 h regeneration period at 37 °C, overexpression was induced with 1 mM IPTG.^45^ Cells were harvested, lysed, cleaved and purified as above for the equivalent unlabeled variants.

### Native chemical ligation of synthetic and recombinant HMGN1 segments

#### Ligation of HMGN1_1–65-thioester to HMGN1_66–99_A66C variants (HMGN1_66–99_A66C, HMGN1_66–99_A66C_pS86, HMGN1_66–99_A66C_pS89, or HMGN1_66–99_A66C_pS98)

HMGN1_1–65-MesNa thioester (2 mM) and HMGN1_66–99_A66C variants (4 mM) were dissolved in degassed ligation buffer (6 M GdnHCL, 200 mM NaH_2_PO_4_/Na_2_HPO_4_, 100 mM TCEP, 100 mM methyl thioglycolate, MTG). The pH was adjusted to 7.2, the sample was incubated at 40 °C overnight and ligation progress was monitored by LC-MS and SDS-PAGE. On completion, the reaction mixture was purified by RP-HPLC on a C18 column (Kromasil) with a gradient from 5–65% ACN in water with 0.1% TFA in 30 min. Fractions containing the ligation product were identified by ESI-MS, combined and lyophilized. For desulfurization, the ligation product (1 mM) was dissolved in desulfurization buffer (6 M GdnHCL, 200 mM NaH_2_PO_4_/Na_2_HPO_4_, 200 mM TCEP, 18 mM VA-44, 18 mM *t*-BuSH, pH 6.6) and incubated for 5 h at 40 °C. The reaction was monitored by LC-MS and the desulfurized protein was purified by RP-HPLC on a C18 column (Kromasil) with a gradient from 5–45% ACN in water with 0.1% TFA over 40 min.

#### Ligation of HMGN1_1–10-thioester/hydrazide variants (HMGN1_1–10, HMGN1_1–10_acK2, HMGN1_1–10_pS6 or HMGN1_1–10_acK2pS6) to HMGN1_11–99_A11C

HMGN1_1-10-hydrazide (51 mM) was dissolved in 6 M GdnHCL, 200 mM NaH_2_PO_4_/Na_2_HPO_4_, 28 mM NaNO_2_, pH 3 and stirred at −15 °C for 15 min to convert the acyl hydrazide to the corresponding azide. HMGN1_11–99_A11C (5.5 mM) was dissolved in 100 μL ligation buffer (6 M GdnHCL, 200 mM NaH_2_PO_4_/Na_2_HPO_4_, 100 mM TCEP, 200 mM MTG), mixed with the hydrazide solution and the ligation mixture was brought to room temperature and adjusted to pH 7.3. Concentrations in the ligation mixture were HMGN1_1–10-thioester 18 mM and HMGN1_11–99_A11C 3.5 mM. The reaction was shaken at room temperature overnight and monitored by LC-MS and SDS-PAGE. The ligation product was purified by RP-HPLC on a C18 column (Kromasil) with a gradient from 5–45% ACN in water over 30 min. Desulfurization was performed by dissolving the ligation product (2 mM) in desulfurization buffer (6 M GdnHCl, 200 mM NaH_2_PO_4_/Na_2_HPO_4_, 200 mM TCEP, 18 mM VA-44, 18 mM *t*-BuSH, pH 6.6) and incubating at 40 °C for 7 h. Desulfurized product was purified by RP-HPLC on a C18 column (Kromasil) with a gradient from 5–65% ACN in water with 0.1% TFA over 30 min.

### NMR data collection and processing

Samples for NMR data collection were prepared in 20 mM Na_2_HPO_4_ buffer with 5% v/v D_2_O (Cambridge Isotope Laboratories, DLM-4-99-100), 10 μM 2,2-dimethyl-2-silapentane-5-sulfonate sodium salt (DSS) as an internal reference,^46^ and 0.02% w/v NaN_3_ at pH 6.0. NMR spectra were acquired at the University of Vienna NMR Centre on an Avance III HDX 700 MHz NMR spectrometer (Bruker BioSpin, Germany) equipped with an inverse helium cooled quadruple cryoprobe (QCI-F) or at the University of Queensland, Centre for Advanced Imaging on an Avance III HD 700 MHz NMR spectrometer equipped with a cryoprobe or an Avance III HD 900 MHz NMR spectrometer equipped with a cryoprobe and all spectra were acquired at 298 K. Spectra acquired for ^15^N/^13^C-labeled unmodified HMGN1 included ^1^H–^15^N HSQC; HNCO; HNCA; HN(CA)CB; HN(CO)CA; HN(CO)(CA)CB; HN(CA)CO; HN(CO)(CA)(N)NH; HN(CA)(N)NH. Spectra acquired for ^15^N-segmentally labeled modified/unmodified HMGN1 segments included ^1^H–^15^N HSQC and ^1^H–^15^N heteronuclear NOE experiments. Spectra acquired for unlabeled HMGN1 segments and random coil peptides included ^1^H–^15^N HSQC; ^1^H–^1^H TOCSY with an isotropic mixing time of 80 ms; ^1^H–^1^H NOESY with a mixing time of 300 ms; ^1^H–^15^N HSQC; and ^1^H–^13^C HSQC. Spectra were Fourier transformed, phased and calibrated on the DSS signal (^1^H at 0 ppm) in Topspin 4.0.6 (Bruker BioSpin, Germany). ^15^N and ^13^C spectra were calibrated on the unified scale according to the IUPAC recommendations,^46,47^ using a ratio of 0.251449530 for ^13^C and 0.101329118 for ^15^N. Spectra were assigned in CCP-NMR v2 and v3.^48^ Secondary chemical shifts were calculated by subtraction of the respective random coil chemical shift from the observed chemical shift.^49,50^ Chemical shift perturbation values for NH chemical shifts were calculated using the formula:^51–53^
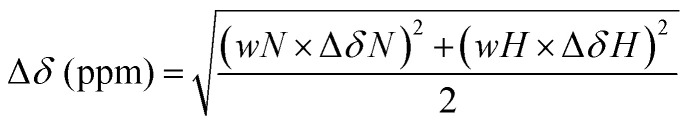
where the weighting values are *w*_N_ = 0.158 and *w*_H_ = 1, and Δ*δN* and Δ*δH* indicate the difference in chemical shift (ppm) between full-length recombinant HMGN1 and the various semi-synthetic and segmentally-labeled HMGN1 variants.

Steady-state ^1^H–^15^N nuclear Overhauser effect (NOE) data were acquired for the uniformly- and segmentally-labelled HMGN1 variants at 700 and 900 MHz.^54^^1^H–^15^N NOE ratios (*I*_NOE_/*I*_st_) were calculated from duplicate pairs of ^1^H–^15^N spectra acquired in an interleaved fashion with and without proton saturation. Phasing and scaling parameters were equal for both spectra and peak heights were calculated in CCPNMR v3.

To investigate the effects of charge screening, ^1^H–^15^N HSQC spectra were acquired as above on HMGN1 variants and N- and C-terminal peptide segments at 298 K on an Avance III HD 900 MHz NMR spectrometer equipped with a cryoprobe. Samples were dissolved in NMR buffer as above, 25 mM NaCl + 25 mM KCl were added and pH was adjusted to 6.0. After acquiring these spectra, salt concentration was increased to 100 mM NaCl + 100 mM KCl, pH was re-adjusted to 6.0 and spectra were acquired again.

### Circular dichroism

Circular dichroism spectra were recorded on a Chirascan Plus CD spectrometer (Applied Photonics, UK) in micro-cuvettes with a 1 mm pathlength at 25 °C. Scans were carried out from 260 to 185 mm with five repetitions. Spectra were examined individually and then averaged and a baseline (H_2_O) spectrum was subtracted. The HMGN1 sample was prepared in H_2_O at a concentration of 5 μM at pH 5.0.

### *In vitro* nucleosome core assembly and electrophoretic mobility shift assays (EMSAs)

Nucleosome cores were assembled onto a 147 bp AlexaFluor488 labelled DNA fragment containing the 601 nucleosome positioning sequence.^55,56^ The AlexaFluor488-labeled fragment was generated by PCR and purified as described previously.^57^ Recombinant *Xenopus laevis* histone octamers were used to assemble nucleosomes, and were produced using standard protocols.^58^ A representative native PAGE gel of the nucleosome preparation is shown in ESI,† Fig. S9.4.

Assembly of nucleosome cores was performed by salt-gradient dialysis as described.^58,59^ HMGN1 variants (2–800 nM) were incubated with AlexaFluor488-labeled nucleosome cores (50 nM) or DNA (50 nM) in gel shift buffer (10 mM Tris pH 7.5, 25 mM NaCl, 25 mM KCl, 0.2 mM DTT, 4% sucrose) on ice for 20–30 min. Binding reactions were loaded onto 5% (w/v) native PAGE gels and electrophoresed in 0.5× TBE at 75 V for 45–90 min at 4 °C. Gels were visualized on a Typhoon™ FLA9000 laser scanner (GE Healthcare).

## Results and discussion

### Characterisation and assignment of unmodified HMGN1

**Fig. 2:**
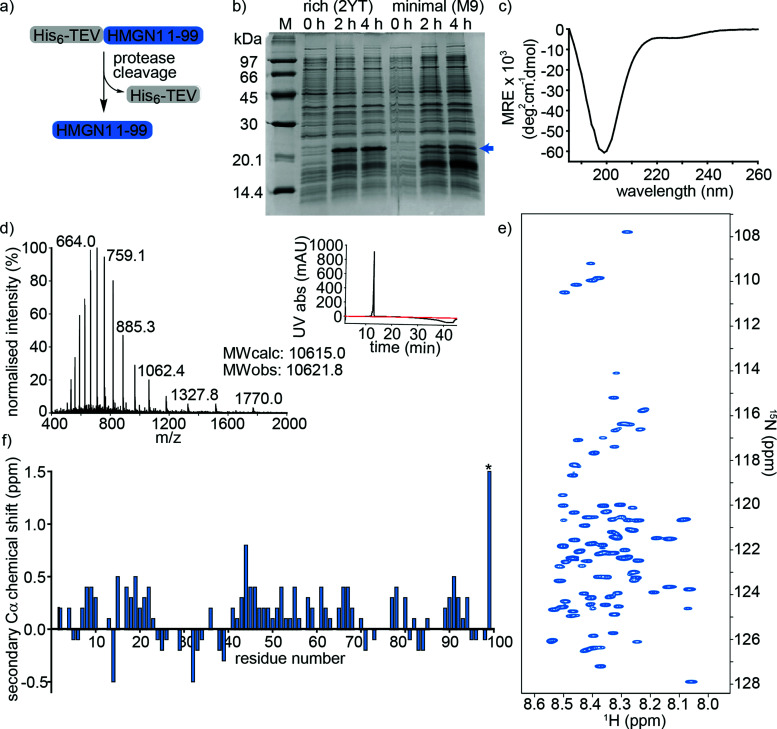
Expression and characterization of full-length unmodified HMGN1. (a) HMGN1 fused at the N-terminus to a His_6_-tag and TEV protease cleavage site was expressed and purified from *E. coli*. Cleavage with TEV protease and RP-HPLC purification yielded full-length HMGN1. (b) SDS-PAGE gel showing expression profile (hours after induction) of His_6_-TEV-HMGN1 construct in rich medium (2YT) and minimal (M9) medium used for ^15^N/^13^C isotope labelling. The blue arrow indicates the position of the His_6_-TEV-HMGN1 protein, which runs at an apparent *M*_W_ of ∼22 kDa (Calculated MW of His_6_-TEV-HMGN1 construct: 12.4 kDa). (c) Circular dichroism (CD) spectrum of purified HMGN1. (d) ESI Mass spectrum of purified HMGN1 with analytical HPLC trace (inset) at 214 nm (black) and 280 nm (red). (e) ^1^H–^15^N HSQC NMR spectrum of uniformly labelled, unmodified HMGN1 showing the narrow dispersion of amide resonances. Residue assignments are labelled in Fig. S6.1 (ESI†) (f) secondary Cα chemical shift plot of unmodified HMGN1. The secondary shift for reside D99 (marked with *) is 1.59 ppm. Secondary Cβ and C′ chemical shift plots are shown in Fig. S8.1 and 8.2 (ESI†).

HMGN1_1–99, preceded by a hexa-histidine tag and TEV-protease cleavage site was expressed in *E. coli* and purified on a NiNTA (affinity) column from the soluble fraction after cell lysis. Cleavage by TEV protease removed the hexa-histidine tag (Fig. 2a), leaving an N-terminal serine preceding the native HMGN1 sequence (Sequence in Table S2, ESI†). This serine residue is part of the recognition sequence (ENLYFQ|S/G) for TEV protease that remains after cleavage and is designated as Ser0 in this work to preserve consistency of residue numbering with HMGN1 sequences in the literature and UniProt databank. The cleaved protein was then purified by HPLC, giving a final yield of ∼1.1 mg L^−1^ of culture. The desired molecular weight of 10.6 kDa was confirmed by mass spectrometry, however HMGN1 runs abnormally on SDS-PAGE, showing an apparent molecular weight of ∼22 kDa (Fig. 2b and d), likely due to its high content of charged residues and disordered nature.^10,60^ The lack of characteristic alpha-helix or beta-sheet maxima and minima in the CD spectrum (Fig. 2c) also gives evidence for the absence of secondary structural features.

Uniform ^15^N- and ^15^N/^13^C-labeling of HMGN1 for NMR spectroscopy was achieved with ∼93% labelling efficiency using a two-step expression protocol in which the *E. coli* cell mass was grown up in rich medium, the cells were pelleted by centrifugation and then resuspended in minimal medium containing isotope labelled nutrients for expression. As shown in Fig. 2b, expression yield and purity was reduced compared to rich medium, but sufficient quantities of uniformly ^15^N/^13^C-labeled HMGN1 (∼0.8 mg L^−1^ of rich medium, 250 mL minimal medium) were obtained to acquire NMR data that enabled assignment of the backbone resonances (Supplementary Data Table S8.1, ESI†). In agreement with the CD data, the limited dispersion of the amide proton chemical shifts confirms the lack of secondary structure; all amide proton shifts lie between 8.0 and 8.6 ppm (Fig. 2e and Fig. S6.1, ESI†). Three-dimensional HNCO, HNCA, HN(CA)CB, HN(CO)CA, HN(CO)(CA)CB, HN(CA)CO, HN(CO)(CA)(N)NH, and HN(CA)(N)NH spectra were acquired on a 700 MHz NMR spectrometer equipped with a cryoprobe and showed sharp signals typical of intrinsically disordered proteins. Some ambiguities caused by overlap of amide resonances were resolved by comparison with spectra of semi-synthetic segmentally-labelled HMGN1 variants generated as described below, illustrating advantages of segmental labelling techniques. Secondary Cα chemical shifts are shown in Fig. 2f and, except for the C-terminal aspartic acid residue, are all <1 ppm, in agreement with an absence of secondary structural features. Some stretches of positive secondary chemical shifts (*e.g.* residues 14–23 and residues 41–71), however might indicate an alpha-helical propensity. Secondary chemical shifts for Cβ and C′ are shown in ESI,† Fig. S8.2 and S8.3 and, similarly to those for Cα, are <1 ppm, indicating a lack of secondary structure. The overall dispersion of the signals and magnitudes of the Cα secondary chemical shifts of HMGN1 are similar to those of HMGN2,^26^ however the latter were assigned for HMGN2 bound to nucleosomes, and show mostly negative secondary Cα chemical shifts.

### N-terminal PTMs of HMGN1: semi-synthesis, segmental labelling and NMR spectroscopy

**Fig. 3:**
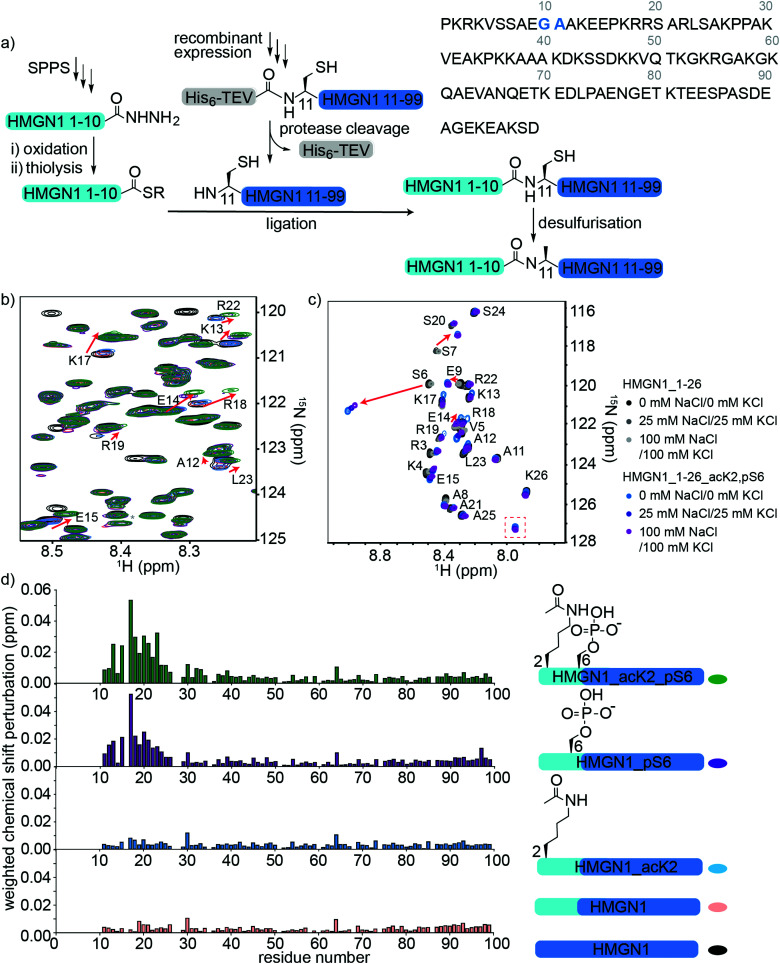
Semi-synthesis and NMR of HMGN1 bearing N-terminal PTMs. (a) Semi-synthesis strategy involving synthesis of the N-terminal segment HMGN1_1–10 (and variants bearing PTMs) by solid phase peptide synthesis (SPPS) and recombinant expression of the C-terminal segment HMGN1_11–99 (with or without uniform ^15^N isotope labeling). Native chemical ligation followed by desulfurization of cysteine to alanine yielded site-specifically modified and segmentally labelled variants of HMGN1. The sequence of HMGN1 is shown with the selected ligation junction in blue. (b) Section of ^15^N-HSQC NMR spectrum overlay of segmentally labelled HMGN1 variants showing resonances in the labelled section that shift when N-terminal PTMs are present (red arrows). Spectra are shown for HMGN1_15N13C (black), HMGN1_unmodN_15N (pink), HMGN1_acK2_15N (blue), HMGN1_pS6_15N (purple) and HMGN1_acK2, pS6 (green), corresponding to the schematic structures in (d). Full spectra and expanded view with all residues labelled are shown in Supplementary Data S6.2 (ESI†). (c) Section of ^15^N-HSQC NMR spectrum overlay of synthetic HMGN1_1–26 (black, dark grey and light grey) and HMGN1_1–26_acK2, pS6 (light blue, dark blue and purple) HMGN1 segments, acquired at natural abundance with increasing salt concentrations. Resonances that shift on introduction of PTMs are shown by red arrows and the new amide resonance from acK2 is shown in a red box. Full spectra are shown in Supplementary Data S6.4 (ESI†). (d) Chemical shift perturbation plots for the segmentally-labelled HMGN1 variants bearing N-terminal PTMs, as shown in (b). Light blue segments are synthetic and unlabelled, dark blue segments are ^15^N isotope-labelled.

To access HMGN1 variants bearing the N-terminal PTMs acetylation at Lys2 (acK2) and/or phosphorylation at Ser6 (pS6), we designed a protein semi-synthesis strategy involving ligation of a synthetic N-terminal segment (residues 1–10) bearing the desired PTMs to a recombinantly expressed C-terminal segment (residues 11–99), which could be expressed in labelled or unlabelled form (Fig. 3a). In the synthetic segment, we can incorporate authentic phosphoserine and acetyl lysine residues using solid phase peptide synthesis (SPPS), in contrast to the alanine, glutamic acid or glutamine mutations widely used for recombinant proteins. As HMGN1 contains no native cysteine residues, we chose a ligation junction for native chemical ligation between residues 10 and 11 to install the N-terminal PTMs. In this strategy, Gly10 at the C-terminus of the synthetic fragment serves as a sterically unhindered acyl donor and Ala11 is mutated to cysteine for ligation. After ligation, the cysteine can be desulfurized using established methods to restore the native HMGN1 sequence.^61^ Synthetic HMGN1_1–10 segments were synthesized by SPPS on a hydrazide resin to yield acyl hydrazides as thioester precursors,^44^ and posttranslationally modified residues were installed using commercially-available protected building blocks. Cleavage, deprotection and HPLC purification yielded the peptide hydrazides HMGN1_1–10, HMGN1_1–10_acK2, HMGN1_1–10_pS6 and HMGN1_1–10_acK2, pS6 (see Supplementary Data S4, ESI† for chemical structures, yields, mass spectra and analytical HPLC data). The C-terminal recombinant segment was expressed in *E. coli* with the A11C mutation, preceded by a His_6_ tag and TEV-protease cleavage site. After purification, TEV-protease cleavage proceeded to ∼80% completion and purification by HPLC yielded the N-terminal cysteine-bearing protein segment (Fig. 3a, yield ∼1.2 mg L^−1^ of rich medium, 1.5 L minimal medium). For ligation of the two HMGN1 segments, *in situ* conversion of the acyl hydrazides to acyl thioesters was achieved by oxidation of the hydrazide to an azide and thiolysis with methyl thioglycolate (MTG).^44^ On addition of the HMGN1_11–99_A11C segment and pH adjustment, the ligation proceeded to completion within 5 h at 40 °C. Although we had selected the alkyl thiol additive MTG for ligation, envisaging a one-pot desulfurization strategy,^39,62^ we found the desulfurization did not go to completion and the ligation product degraded over time. However, HPLC purification of the ligation product prior to desulfurization gave cleaner and more complete semi-synthetic HMGN1 variants with native sequences and the desired PTMs (see Supplementary Datas S2 and S3, ESI† for sequences, yields, mass spectra and analytical HPLC data). Ligation of uniformly ^15^N-labelled HMGN1_11–99 segments yielded the corresponding site-specifically modified and segmentally labelled HMGN1 variants.

Comparison of ^15^N-HSQC spectra of segmentally labelled unmodified HMGN1 with unmodified uniformly labelled HMGN1 (Fig. 3b and Fig. S6.1, ESI†) shows that they have identical NMR ‘fingerprints’ and that the ligation-desulfurization protocol does not alter the structural properties of the protein. As the synthetic segment is unlabelled, backbone amide resonances of residues 1–10 are absent in the ^15^N-HSQC spectrum of segmentally-labelled HMGN1, which was helpful for confirming assignments of these residues. ^15^N-HSQC spectra of HMGN1 variants bearing PTMs in the synthetic, unlabelled segment show small shifts in several amide resonances within the labelled segment (Fig. 3b, expanded view in ESI,† Fig. S6.2). This is significant because it shows that even small PTMs, such as addition of a phosphate or acetyl group to a side chain, can cause long-range perturbations in the chemical environment of backbone residues that are distant from the modification site, even in a disordered protein. As shown in the chemical shift perturbation plots in Fig. 3d, acK2 alone did not result in any shifts of amide resonances in the labelled segment, however, the HMGN1 variant bearing pS6 displayed shifts in residues 14–24, which fall in the nucleosome-binding domain. These shifts are larger in the HMGN1 variant bearing both acK2 and pS6 PTMs. Shifts in the same residues with slightly decreased magnitude were observed for the HMGN1 variants when spectra were acquired in buffer containing 25 mM NaCl and 25 mM KCl, corresponding to salt concentrations used in the electrophoretic gel assays (ESI,† Fig. S6.3). This observation confirms that these residues are influenced by the PTMs even under altered conditions and supports that the interactions of the PTMs with other residues are likely to be electrostatic in nature. The changes in shift are more clearly seen in the less-crowded spectra of the HMGN1_1–26 segments as shown in Fig. 3c.

Although HMGN2 (89 residues) is shorter than HMGN1 (99 residues) and differs in other domains, the nucleosome-binding domains (NBDs) of both proteins are conserved. In a study of HMGN2 binding to nucleosomes, Kato *et al.* proposed that residues 22–28 bind to the acidic patch of nucleosomes and that phosphorylation of Ser24 and Ser28 in HMGN2 (mimicked by mutation to glutamic acid) disrupts electrostatic interactions with the acidic patch.^26^ Although we did not investigate the effects of PTMs in the NBD in this study, our results suggest that in addition to potential direct electrostatic interactions, PTMs in the N-terminus might also influence the conformation and binding of the NBD. This model is consistent with that proposed by Lim *et al.*, who hypothesized that HMGN1 hinders access of mitogen-activated kinases MSK1 and MSK2 to histone H3.^63^ Phosphorylation of HMGN1 at Ser6 by these kinases led to reduced binding to nucleosomes, thereby allowing access of MSK1 and MSK2 to histone H3 and phosphorylation of H3 Ser10.^63^

To verify these long-range perturbations and explore any other shorter-range changes, HMGN1_1–26 segments, either unmodified, or bearing both acK2 and pS6 PTMs, were synthesized by SPPS (Supplementary Data S3, ESI†). These segments are unlabelled because they are obtained by SPPS, but homonuclear TOCSY and NOESY spectra and ^15^N-HSQC spectra were acquired at natural abundance and were sufficient for backbone assignment for this 26-residue segment. (NMR spectroscopy of full-length HMGN1 (99 residues) at natural ^15^N/^13^C abundance would be impractical because of significant signal overlap in the homonuclear spectra and high concentrations required to compensate for low sensitivity at natural abundance). Hence, segmental labelling strategies as described above were used for the full-length HMGN1 variants. The spectra shown in Fig. 3c and Fig. S6.4 (ESI†) show characteristic chemical shift changes of the modified residues as predicted by their random coil shifts,^50^ namely a downfield shift (0.51 ppm) of the amide proton chemical shift upon phosphorylation and appearance of a new amide resonance at (7.95 pm/127.2 ppm) from the amide moiety in acetyllysine (Fig. 3c), in agreement with literature values.^50^ In addition to shifts in the resonances of residues flanking the PTMs, shifts were also seen in the more distant residues 12–20, corresponding to those seen for full-length segmentally-labelled HMGN1_acK2_pS6 (Fig. 3b). Although the shifts in residues 12–20 on modification of Lys2 and Ser6 suggest an interaction between these two regions, no new NOESY peaks were observed that would indicate an overall structural change or formation of new secondary structure elements. However, weak transient interactions are not expected to give rise to ^1^H–^1^H NOEs between disordered protein regions.

To investigate the possibility of restricted motion caused by interactions of PTMs with regions of HMGN1 that are distant in primary sequence, we measured heteronuclear ^1^H–^15^N nuclear Overauser effect (NOE) ratios at 700 MHz and 900 MHz for unmodified HMGN1 and the HMGN1 variant bearing both acK2 and pS6 PTMs (Supplementary Data S7.1, ESI†).^54,64^ As expected, NOE ratios increase with increasing magnetic field strength and the majority of the NOE ratios were close to zero, reflecting the intrinsic disorder of HMGN1. NOE ratios for the N- and C-termini of HMGN1 are more negative than those of the central regions, corresponding to greater flexibility of the termini, particularly residues 1–30 and 90–99. The PTMs acK2 and pS6, however, do not appear to alter the flexibility of HMGN1, which would be indicated by an increase or decrease in the NOE ratio for a region of the protein. Similarly, comparison of the NOE ratios of the other modified variants HMGN1_pS6, HMGN1_pS88, and HMGN1_pS85, 88, 98 did not reveal any differences in motion compared to unmodified HMGN1 (Supplementary Datas S7.2 and S7.3, ESI†).

### C-terminal PTMs of HMGN1: semi-synthesis, segmental labelling and NMR spectroscopy

**Fig. 4:**
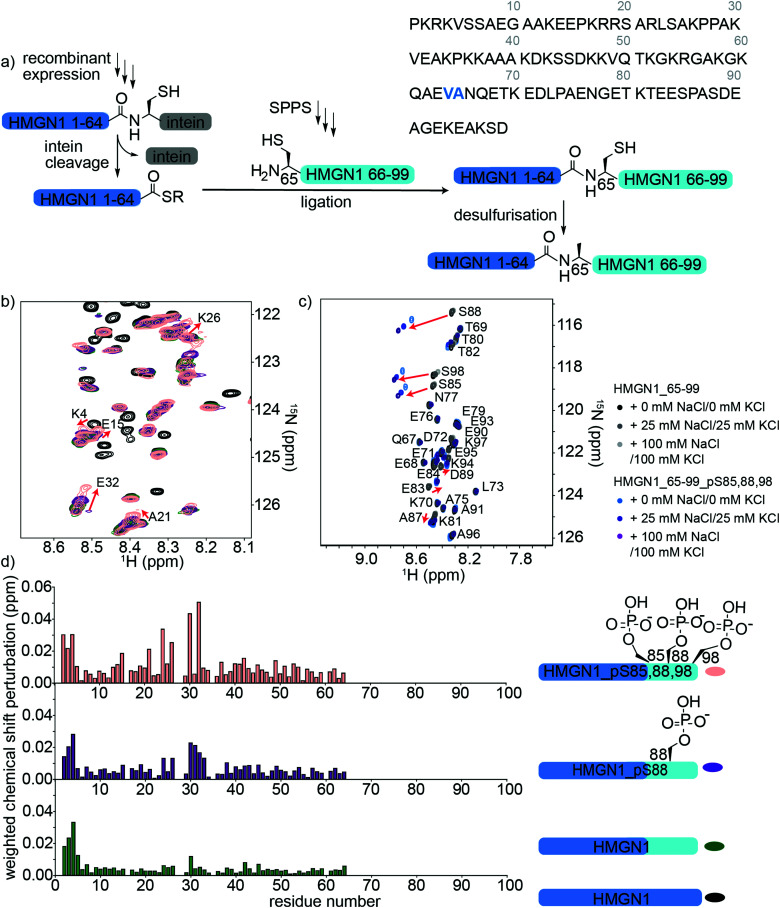
Semi-synthesis and NMR of HMGN1 bearing C-terminal PTMs. (a) Semi-synthesis strategy involving recombinant expression of the N-terminal segment HMGN1_1–64 (with or without uniform ^15^N isotope labelling) and synthesis of C-terminal segment HMGN1_65–99_A65C (and variants bearing PTMs) by solid phase peptide synthesis (SPPS). Native chemical ligation followed by desulfurization of cysteine to alanine yielded site-specifically modified and segmentally labelled variants of HMGN1. The sequence of HMGN1 is shown with the selected ligation junction in blue. (b) Section of ^15^N-HSQC NMR spectrum overlay of segmentally labelled HMGN1 variants showing resonances in the labelled section that shift when C-terminal PTMs are present (red arrows and residue labels in black). Spectra are shown for HMGN1_15N13C (black), HMGN1_unmodC_15N (green), HMGN1_pS88_15N (purple), HMGN1_pS85, 88, 98_15N (pink), corresponding to the schematic structures in (d). Full spectra and expanded view with all residues labelled are shown in Supplementary Data S6.5 (ESI†). (c) Section of ^15^N-HSQC NMR spectrum overlay of synthetic HMGN1_65–99 (black, dark grey and light grey) and HMGN1_65–99_pS85, 88, 98 (light blue, dark blue and purple), acquired at natural abundance with increasing salt concentrations. Resonances that shift on introduction of PTMs are shown by red arrows. Full spectra and spectra of HMGN1_65–99_pS85 and HMGN1_65–99_pS8 are shown in Supplementary Datas S6.6 and S6.7 (ESI†). (d) Chemical shift perturbation plots for the segmentally-labelled HMGN1 variants bearing C-terminal PTMs, as shown in (b). Light blue segments are synthetic and unlabelled, dark blue segments are ^15^N isotope labelled.

HMGN1 variants bearing the C-terminal PTMs pS85, pS88, pS98 were accessed *via* an expressed protein ligation (EPL) semi-synthesis strategy, as shown in Fig. 4a.^65^ The N-terminal segment HMGN1_1–64 was expressed in *E. coli* fused to the Mxe GyrA intein, followed by a His_7_ tag and chitin binding domain (sequence in Supplementary Data S1, ESI†). The fusion protein was expressed in inclusion bodies and was resolubilized and purified under denaturing conditions. Buffer exchange to urea and dilution of the denaturant in the presence of the thiol sodium mercaptoethane sulfonate (MesNa) led to folding and cleavage of the intein to yield the N-terminal segment of HMGN1 bearing a C-terminal thioester, which was purified by HPLC from the cleaved intein and a small amount of uncleaved fusion protein (Supplementary Data S5, ESI†). Three variants of the C-terminal segment HMGN1_65–99 were synthesized by SPPS with residue 65 as a cysteine in place of the native alanine (sequences, yields, mass spectra and analytical HPLC are shown in the Supplementary Datas S2 and S3, ESI†). This mutation allows for native chemical ligation and subsequent desulfurization to yield the native sequence, as for the N-terminally modified variants. Despite the choice of β-branched valine as the acyl donor residue in our strategy, ligation proceeded to completion within 5 h at 40 °C and subsequent desulfurization yielded three HMGN1 variants: unmodified, pS88 and pS85, 88, 98 (Supplementary Data S3, ESI†). Corresponding segmentally labelled variants were also prepared by expression of the N-terminal intein fusion segment in ^15^N-labeled medium.

As for the N-terminal ligation strategy, comparison of the ^15^N HSQC spectra of segmentally labelled unmodified HMGN1 with that of fully labelled HMGN1 showed that our semi-synthesis strategy did not change the conformation of unmodified HMGN1 (Supplementary Data S6.5, ESI†). Resonances for residues 65–99 absent in the spectra of segmentally labelled HMGN1 aided in assignment of fully labelled HMGN1. ^15^N-HSQC spectra of the variants bearing pS85 and pS85, 88, 98 PTMs showed shifts and broadening of several signals, indicating changes in equilibrium conformations, as for the N-terminal PTMs. As shown in Fig. 4d, the resonances most affected by the phosphorylation PTMs lie in the region 24–34, which is within the nucleosome binding domain. Larger changes for the triply-modified variant HMGN1_pS85, 88, 98 compared to the singly-modified variant suggest that the PTMs have a cumulative effect on the conformational preferences. In contrast to the shifts observed on the introduction of N-terminal PTMs, where signals in a region shifted to a new position, the C-terminal phosphorylations caused peaks to broaden, as exemplified by Glu32 (Fig. 4b, expanded view in ESI,† Fig. S6.5). This indicates that several conformations are present and interchanging on the NMR timescale. Furthermore, the region of residues affected is more dispersed than that observed for the N-terminal PTMs (Fig. 3b). Whereas several studies have focused on phosphorylations in the NBD, the role of phosphorylation in the C-terminal regulatory domain is less well characterized. Phosphorylation of Ser6, Ser85, Ser88 and Ser98 was detected in HMGN1 isolated from MCF-7 breast cancer cells and it was noted that phosphorylation would introduce even more negative charges in this region, which is already highly negatively charged.^66^

In the NMR spectra of the synthetic unlabelled HMGN1_65–99 segments (Fig. 4c and S6.6, S6.7, ESI†), downfield shifts in the backbone amide resonances of the phosphorylated residues were observed as expected for pSer. However, the changes in chemical shift upon phosphorylation relative to the unmodified residue differ depending on the sequence position; whereas Ser85 shifts by 0.21 ppm in the ^1^H dimension, Ser88 shifts by 0.32 ppm, Ser98 shifts by 0.24 ppm, Ser6 shifts by 0.51 ppm (Fig. 4c) and the shift observed for the random coil peptide is 0.32 ppm.^50^ Although HMGN1 is unstructured and most resonances have chemical shifts close to their random coil values (Fig. 2e and f), this indicates that shifts upon phosphorylation are dependent on the sequence context. In the presence of 25 mM NaCl/25 mM KCl and 100 mM NaCl/100 mM KCl (ESI,† Fig. S6.8), the changes in chemical shift of residues flanking the phosphorylated residues are the same, while those of the phosphorylated residues increase. This is in contrast to the slight decrease in chemical shift perturbation of the phosphorylated residue in the N-terminal segment and might be rationalised by the different sequence context; whereas phosphorylation of Ser6 introduces negative charge into an overall positively-charged region, phosphorylation of Ser85, 88 and 98 introduce negative charge into an already highly negatively charged region, likely increasing repulsion and chain extension.

We hypothesized that the changes in conformational equilibria observed on phosphorylation might be caused by a shift in proline *cis*/*trans* isomer proportions, especially as HMGN1 contains several proline residues. In particular, the PTM pS85 precedes a proline residue and we explored whether serine phosphorylation might alter the *cis*/*trans* proline ratio, as has been reported for some proteins such as those which are substrates of the peptidyl-prolyl *cis*/*trans* isomerase Pin1.^42,67^ This possibility was also suggested when Ser85 phosphorylation was first detected,^66^ but was not further investigated. We observed a very low intensity signal close to the amide resonance of Ser88, which might originate from a minor *cis*-Pro population, however it was too weak to assign and was not observed for the corresponding modified peptide. Using short model peptides derived from those used to determine the random coil shifts of posttranslationally modified residues,^50^ we compared the proportion of *cis*- and *trans*-proline conformations when preceded by serine or phosphoserine. As shown in the Supplementary Data (S6.8) (ESI†), although characteristic shifts are observed for phosphoserine, we did not observe any significant change in the proportion of *cis*-proline when preceded by serine (10%) compared to phosphoserine (11%), as determined by comparison of the relative peak volumes. These ratios are similar to those observed for proline flanked by glycine residues in short random coil peptides.^50^ Although, this result does not preclude the possibility that the phosphoserine could be a recognition motif for a peptidyl-prolyl isomerase, the data from the short model peptides, spectra in the presence of salt and the distance in primary sequence of the residues that shift from the phosphoserine residues suggest that conformational effects are more likely to be due to electrostatic interactions.

### Binding of modified and unmodified HMGN1 variants to nucleosome core particles and DNA

**Fig. 5:**
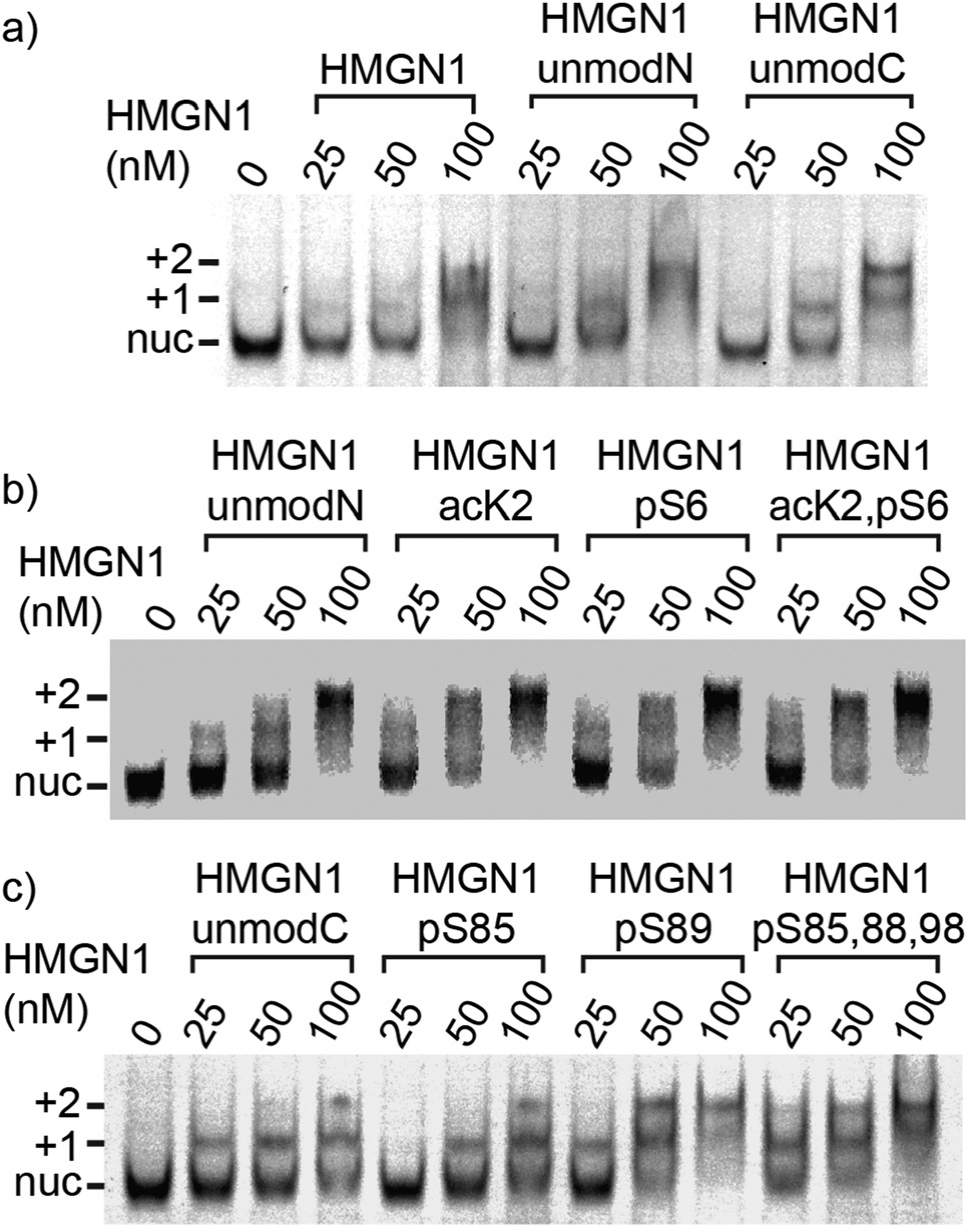
Nucleosome-binding of HMGN1 variants. Electrophoretic mobility shift assays showing mononucleosome binding of HMGN1 variants bearing N- or C-terminal PTMs. Position of nucleosomes bound by one HMGN1 molecules (+1), and two HMGN1 molecules (+2) are shown in relation to the unbound nucleosome (nuc). (a) Mononucleosomes (50 nM) were incubated with full length recombinant or semi-synthetic HMGN1 at molar ratios of 0.5 : 1, 1 : 1 and 2 : 1 HMGN1 molecules per nucleosome. Binding reactions were separated on 5% TBE-acrylamide gels and then scanned for fluorescence. (b) and (c) Comparison of semi-synthetic HMGN1 proteins bearing N- or C-terminal PTMs. HMGN1 variants were incubated with mononucleosomes at molar rations of 0.5 : 1, 1 : 1, and 2 : 1 HMGN1 per nucleosome.

We compared binding of recombinant and semi-synthetic HMGN1 variants and segments to mononucleosomes to see if the conformational changes observed in the nucleosome binding region correspond to functional changes in nucleosome binding. Fig. 5 shows the results of electrophoretic gel mobility assays in which HMGN1 variants were titrated with reconstituted nucleosomes. Overall, the results show that all HMGN1 variants bind to nucleosomes and the major band at 2 : 1 HMGN1 : nucleosome ratios corresponds to two HMGN1 molecules per nucleosome (Fig. 5a), as would be expected from binding of one HMGN1 to each face of the nucleosome. This result agrees with early studies on HMGN1 (previously HMG 14),^68^ however in the nucleus, the number of HMGN1 molecules is thought to be limiting, with 0.5–1.5 molecules per nucleosome.^15,27^ At higher concentrations of HMGN1, additional bands are observed (ESI,† Fig. S9.3), suggesting that more than two HMGN1 molecules can bind per nucleosome.

The electrophoretic mobility shift assays with the N-terminal PTMs (Fig. 5b) show that all the HMGN1 variants bind to nucleosome cores and that there are no major differences in binding of variants. The long-range effects of the PTMs observed in the NMR spectra, some of which are in the nucleosome binding region, therefore do not seem to have a significant effect on binding to nucleosome core particles. The HMGN1 variants with C-terminal PTMs (Fig. 5c) likewise all bind to nucleosomes, and there appears to be a trend of improved binding with the HMGN1_pS89 and HMGN1_pS85, 88, 98 variants. More sensitive assays will be needed to further investigate this trend. Overall, these data suggest that the N- and C-terminal PTMs of HMGN1 affect other functions of HMGN1, such as competition with histones, the formation of high-order chromatin structures, binding to linker DNA, or serving as a docking pad for other chromatin interacting proteins (analogous to the histone code).^1^ Future experiments and more sensitive assays will test these and other possibilities.

Truncated HMGN1 variants in which the N-terminal residues (1–10) or C-terminal residues (65–99) were missing were also compared with full-length HMGN1 for their nucleosome-binding properties, as shown in ESI,† Fig. S9.1. Considering the lower molecular weights, the truncated variants show similar nucleosome binding to full-length HMGN1, showing that the N- and C-terminal regions are not directly involved in nucleosome core binding. These results support the orientation of HMGN1 shown in Fig. 1a, based on that predicted for HMGN2 using molecular modelling techniques.^26^ Experiments in which the HMGN1 variants were mixed with DNA (147 bp AlexaFluor488 labelled DNA containing the 601 nucleosome positioning sequence, Supplementary Data Fig. S9.2, ESI†) indicated that there is no difference in binding of the HNGN1 variants to naked DNA.

In summary, we have generated the first set of HMGN1 variants bearing site-specific PTMs and combinations of PTMs, focusing on the N- and C-terminal PTMs and introducing genuine phosphoserine and acetyllysine residues rather than mimics of these PTMs. We have characterised HMGN1 using NMR spectroscopy and report the chemical shifts of this intrinsically disordered protein. Employing protein semi-synthesis techniques also allowed us to introduce segmental isotope labels and to detect subtle structural changes in regions distant to the modification site; changes that would be difficult to measure without access to specifically-modified variants or using other structural biology techniques that are more suited to structured proteins. For HMGN1, we show that PTMs cause chemical shift changes in the modified residues, as predicted by the random coil shifts, and shifts in their flanking residues. Moreover, N- and C-terminal PTMs of HMGN1 cause long-range chemical shift perturbations in residues within the nucleosome-binding domain but this did not significantly affect the binding to nucleosome cores, although there might be a trend suggesting increased binding in the presence of the C-terminal phosphorylations. These results suggest that the N- and C-terminal PTMs have other biological functions. Overall, this study shows that our approach of combining protein semi-synthesis techniques with structural biology and functional assays allows us to study the effects of specific posttranslational modifications and combinations of modifications, providing mechanistic insights that link protein structure and dynamics with function. Furthermore, understanding the roles of the abundant non-histone components of chromatin and how they regulate chromatin dynamics will help to gain a more complete picture of how gene expression is regulated in health and disease.

## Conflicts of interest

The authors declare no conflict of interest.

## Supplementary Material

CB-002-D0CB00175A-s001
