# Supplementary material for: Site-specific modification and segmental isotope labelling of HMGN1 reveals long-range conformational perturbations caused by posttranslational modifications
Source: RSC Chem Biol. 2021 Jan 5;2(2):537–50. doi: 10.1039/d0cb00175a (PMC8341956; doi:10.1039/d0cb00175a)
Supplement: CB-002-D0CB00175A-s001 [file CB-002-D0CB00175A-s001.pdf]

Electronic Supplementary Material (ESI) for RSC Chemical Biology.
This journal is © The Royal Society of Chemistry 2020

Supplementary Data for

Site-specific modification and segmental isotope labelling of HMGN1 reveals long-range conformational perturbations caused by posttranslational modifications

Gerhard Niederacher[1], Debra Urwin[2], Yasmin Dijkwel,[2] David J. Tremethick[2], K. Johan Rosengren[3], Christian F. W. Becker[1], and Anne C. Conibear[3*]

[1] University of Vienna, Faculty of Chemistry, Institute of Biological Chemistry, Währinger Straße 38, 1090 Vienna, Austria.

[2] The Australian National University, John Curtin School of Medical Research, Department of Genome Sciences, ACT 2601, Australia.

[3] The University of Queensland, School of Biomedical Sciences, Brisbane, QLD 4072, Australia.

[*]Corresponding author:

Anne C. Conibear

The University of Queensland, School of Biomedical Sciences, St Lucia 4072, Brisbane, Australia.

E-mail: a.conibear@uq.edu.au

Phone: + 61-7-3365-1738

Table of Contents

1. DNA and protein sequences for recombinant protein constructs
2. Table of HMGN1 variants generated
3. Mass spectra and analytical HPLC data of HMGN1 variants
4. Structures, mass spectra and analytical HPLC data of synthetic N-terminal HMGN1 segments for ligation
5. Full SDS PAGE gels of protein expression
6. Full NMR spectra
7. Heteronuclear ^{1}H-^{15}N NOE ratios
8. Backbone chemical shift assignments of unmodified HMGN1
9. Gels of nucleosome and DNA binding assays

1. **Gene constructs and sequences**

1.1 Full length HMGN1 (His$_6$-TEV-HMGN1)

DNA sequence:

CAT ATG CAC CAC CAC CAC CAC CAC GAA AAC CTG TAT TTT CAG AGC CCG AAA CGC AAA GTG TCC TCT GCC GAA GGA GCA GCG AAA GAA GAA CCG AAA CGT CGC TCA GCT CGC TTA AGC GCG AAA CCA CCG GCG AAA GTT GAA GCGAAA CCG AAG AAA GCT GCA GCC AAA GAC AAG TCG AGC GAC AAG AAA GTC CAG ACC AAG GGC AAA CGTGGT GCC AAA GGC AAA CAA GCC GAA GTA GCG AAT CAG GAG ACT AAA GAG GAT CTG CCC GCA GAA AACGGG GAA ACG AAA ACC GAA GAG AGT CCT GCA TCG GAT GAA GCT GGT GAG AAG GAA GCG AAA AGC GAT TGA CTC GAG

Amino acid sequence:

MHHHHHHENLYFQSPKRKVSSAEGAAKEEPKRRSARLSAKPPAKVEAKPKKAAAKDK SSDKKVQTKGKRGAKGKQAEVANQETKEDLPAENGETKTEESPASDEAGEKEAKSD

1.2 His$_6$-TEV-HMGN1_11-99

DNA sequence:

CAT ATG CAT CAC CAT CAC CAC CAT GAA AAT CTG TAT TTT CAG TGT GCT AAA GAG GAG CCT AAG CGT CGT TCG GCT CGT TTG AGT GCTAAG CCT CCG GCT AAG GTG GAA GCT AAG CCT AAA AAG GCT GCG GCC AAA GAT AAA TCG TCT GAC AAA AAA GTA CAA ACG AAG GGA AAA CGT GGG GCC AAA GGT AAA CAG GCG GAA GTG GCTAAC CAG GAG ACC AAG GAG GAC CTT CCT GCG GAA AAC GGT GAG ACA AAG ACA GAA GAA AGT CCT GCA TCT GAT GAG GCA GGA GAG AAA GAG GCG AAA TCG GAT TGA CTC GAG

Amino acid sequence:

MHHHHHHENLYFQCAKEEPKRRSARLSAKPPAKVEAKPKKAAAKDKSSDKKVQTKGK RGAKGKQAEVANQETKEDLPAENGETKTEESPASDEAGEKEAKSD

1.3 HMGN1_1-65_Mxe_His$_7$_CBD

DNA sequence (Mxe-HIS$_7$-CBD not included):

CAT ATG CCG AAA CGC AAA GTC AGC AGT GCA GAA GGT GCA GCG AAA GAG GAA CCG AAA CGC CGT TCT GCT CGC CTG TCA GCC AAA CCT CCA GCG AAA GTT GAG GCG AAA CCG AAG AAA GCA GCC GCC AAG GAC AAA TCG TCC GAT AAG AAG GTG CAG ACC AAA GGGAAA CGT GGC GCT AAA GGC AAA CAA GCG GAA GTA TGC ATC ACG GGA GAT GCA CTA GT

Amino acid sequence:

PKRKVSSAEGAAKEEPKRRSARLSAKPPAKVEAKPKKAAAKDKSSDKKVQTKGKRGA KGKQAEV(CITGDALVALPEGESVRIADIVPGARPNSDNAIDLKVLDRHGNPVLADRLFH SGEHPVYTVRTVEGLRVTGTANHPLLCLVDVAGVPTLLWKLIDEIKPGDYAVIQRSAFS VDCAGFARGKPEFAPTTYTVGVPGLVRFLEAHHRDPDAQAIADELTDGRFYYAKVASV TDAGVQPVYSLRVDTADHAFITNGFVSHATGLTGIHHHHHHSGLNSGLTTNPGVSAW QVNTAYTAGQLVTYNGKTYKCLQPHTSLAGWEPSNVPALWQLQ)

2. Table of HMGN1 variants generated in this study

Name	MW (Da)	Sequence
HMGN1_S0	10615.0	SPKRKVSSAEGAAKEEPKRRSARLSAKPPAKVEAKPKKAAAKDKSSDKKVQTKG KRGAKGKQAEVANQETKEDLPAENGETKTEESPASDEAGEKEAKSD
HMGN1_S0_15N	10755.8	SPKRKVSSAEGAAKEEPKRRSARLSAKPPAKVEAKPKKAAAKDKSSDKKVQTKG KRGAKGKQAEVANQETKEDLPAENGETKTEESPASDEAGEKEAKSD
HMGN1_S0_15N13C	11200.0	SPKRKVSSAEGAAKEEPKRRSARLSAKPPAKVEAKPKKAAAKDKSSDKKVQTKG KRGAKGKQAEVANQETKEDLPAENGETKTEESPASDEAGEKEAKSD
HMGN1_unmodN	10527.8	PKRKVSSAEGAAKEEPKRRSARLSAKPPAKVEAKPKKAAAKDKSSDKKVQTKGK RGAKGKQAEVANQETKEDLPAENGETKTEESPASDEAGEKEAKSD
HMGN1_unmodN_15N	10652.8	PKRKVSSAEGAAKEEPKRRSARLSAKPPAKVEAKPKKAAAKDKSSDKKVQTKGK RGAKGKQAEVANQETKEDLPAENGETKTEESPASDEAGEKEAKSD
HMGN1_acK2	10569.8	P**K(ac)**RKVSSAEGAAKEEPKRRSARLSAKPPAKVEAKPKKAAAKDKSSDKKVQT KGKRGAKGKQAEVANQETKEDLPAENGETKTEESPASDEAGEKEAKSD
HMGN1_acK2_15N	10694.9	P**K(ac)**RKVSSAEGAAKEEPKRRSARLSAKPPAKVEAKPKKAAAKDKSSDKKVQT KGKRGAKGKQAEVANQETKEDLPAENGETKTEESPASDEAGEKEAKSD
HMGN1_pS6	10607.8	PKRKV**(pS)**SAEGAAKEEPKRRSARLSAKPPAKVEAKPKKAAAKDKSSDKKVQTK GKRGAKGKQAEVANQETKEDLPAENGETKTEESPASDEAGEKEAKSD
HMGN1_pS6_15N	10732.8	PKRKV**(pS)**SAEGAAKEEPKRRSARLSAKPPAKVEAKPKKAAAKDKSSDKKVQTK GKRGAKGKQAEVANQETKEDLPAENGETKTEESPASDEAGEKEAKSD
HMGN1_acK2_pS6	10649.8	P**K(ac)**RKV**(pS)**SAEGAAKEEPKRRSARLSAKPPAKVEAKPKKAAAKDKSSDKKV QTKGKRGAKGKQAEVANQETKEDLPAENGETKTEESPASDEAGEKEAKSD
HMGN1_acK2_pS6_15N	10774.9	P**K(ac)**RKV**(pS)**SAEGAAKEEPKRRSARLSAKPPAKVEAKPKKAAAKDKSSDKKV QTKGKRGAKGKQAEVANQETKEDLPAENGETKTEESPASDEAGEKEAKSD
HMGN1_unmod_C	10527.8	PKRKVSSAEGAAKEEPKRRSARLSAKPPAKVEAKPKKAAAKDKSSDKKVQTKGK RGAKGKQAEVANQETKEDLPAENGETKTEESPASDEAGEKEAKSD
HMGN1_unmod_C_15N	10623.7	PKRKVSSAEGAAKEEPKRRSARLSAKPPAKVEAKPKKAAAKDKSSDKKVQTKGK RGAKGKQAEVANQETKEDLPAENGETKTEESPASDEAGEKEAKSD

HMGN1_pS85	10607.8	PKRKVSSAEGAAKEEPKRRSARLSAKPPAKVEAKPKKAAAKDKSSDKKVQTKGK RGAKGKQAEVANQETKEDLPAENGETKTEE**(pS)**PASDEAGEKEAKSD
HMGN1_pS88	10607.8	PKRKVSSAEGAAKEEPKRRSARLSAKPPAKVEAKPKKAAAKDKSSDKKVQTKGK RGAKGKQAEVANQETKEDLPAENGETKTEESPA**(pS)**DEAGEKEAKSD
HMGN1_pS88 _15N	10703.6	PKRKVSSAEGAAKEEPKRRSARLSAKPPAKVEAKPKKAAAKDKSSDKKVQTKGK RGAKGKQAEVANQETKEDLPAENGETKTEESPA**(pS)**DEAGEKEAKSD
HMGN1_pS85 ,88,98	10767.8	PKRKVSSAEGAAKEEPKRRSARLSAKPPAKVEAKPKKAAAKDKSSDKKVQTKGK RGAKGKQAEVANQETKEDLPAENGETKTEE**(pS)**PA**(pS)**DEAGEKEAK**(pS)**D
HMGN1_pS85 ,88,98_15N	10863.6	PKRKVSSAEGAAKEEPKRRSARLSAKPPAKVEAKPKKAAAKDKSSDKKVQTKGK RGAKGKQAEVANQETKEDLPAENGETKTEE**(pS)**PA**(pS)**DEAGEKEAK**(pS)**D
HMGN1_1-65	6810.0	PKRKVSSAEGAAKEEPKRRSARLSAKPPAKVEAKPKKAAAKDKSSDKKVQTKGK RGAKGKQAEV
HMGN1_11- 99	9487.6	AAKEEPKRRSARLSAKPPAKVEAKPKKAAAKDKSSDKKVQTKGKRGAKGKQAE VANQETKEDLPAENGETKTEESPASDEAGEKEAKSD
HMGN1_65- 99	3767.8	CNQETKEDLPAENGETKTEESPASDEAGEKEAKSD
HMGN1_65- 99_pS88	3847.8	CNQETKEDLPAENGETKTEESPA**(pS)**DEAGEKEAKSD
HMGN1_65- 99_pS85	3847.8	CNQETKEDLPAENGETKTEE**(pS)**PASDEAGEKEAKSD
HMGN1_65- 99_pS85,88,98	4007.8	CNQETKEDLPAENGETKTEE**(pS)**PA**(pS)**DEAGEKEAK**(pS)**D
HMGN1_1-26	2838.3	PKRKVSSAEGAAKEEPKRRSARLSAK
HMGN1_1- 26_acK2,pS6	2960.3	P**K(ac)**RKV**(pS)**SAEGAAKEEPKRRSARLSAK
Random coil_SP	472.5	Ac-GGSPGG-NH_2
Random coil_pSP	551.5	Ac-GG**(pS)**PGG-NH_2

K(ac) = *N*-acetyllysine, **(pS)** = phosphoserine, Ac = N-terminal acetylation

Residues in blue are ^{15}N-labelled, residues in green are ^{15}N/^{13}C-labelled.

3. Mass spectra and analytical HPLC analyses of HMGN1 variants

ESI mass spectra were obtained in positive ion mode and intensities are normalised to the highest intensity peak. Analytical RP-HPLC was carried out using a C4 analytical HPLC column and a 2%/min gradient of acetonitrile (0.045% TFA) in water (0.05% TFA). UV absorbance was detected at 214 nm (black traces) and 280 nm (red traces).

HMGN1_S0 (Yield after expression, purification, TEV cleavage and HPLC purification: ~ 1.1 mg/L of culture, 1.6 mg)

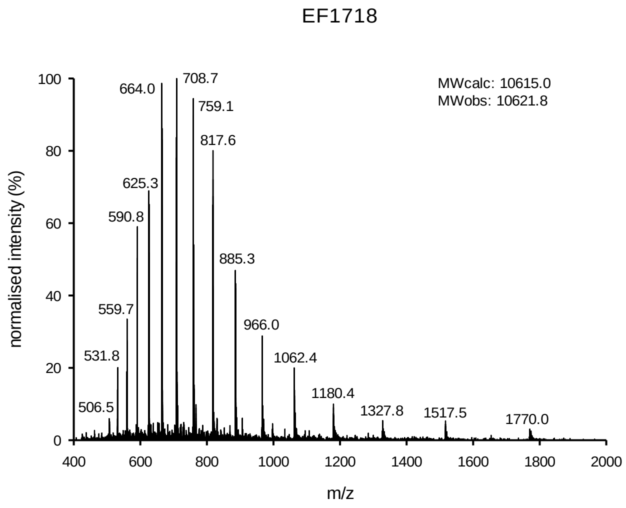

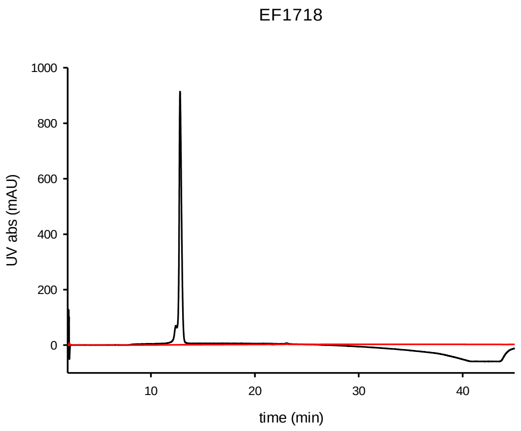

HMGN1_S0_15N (Yield after expression, purification, TEV cleavage and HPLC purification: ~ 1 mg/L of rich medium, 250 mL minimal medium, 1.2 mg)

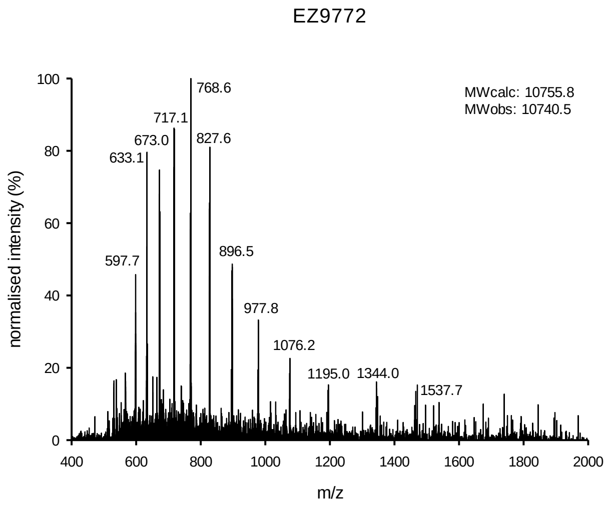

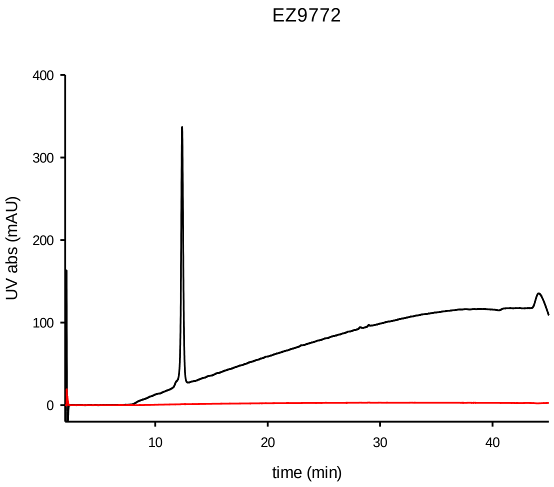

HMGN1_S0_15N13C (Yield after expression, purification, TEV cleavage and HPLC purification: ~ 0.8 mg/L of rich medium, 250 mL minimal medium, 1.7 mg)

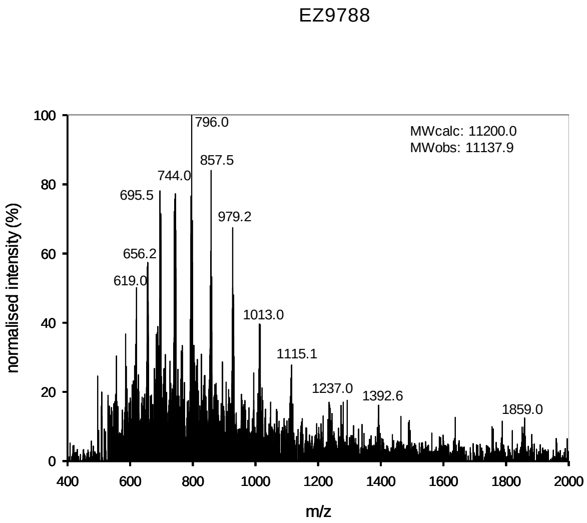

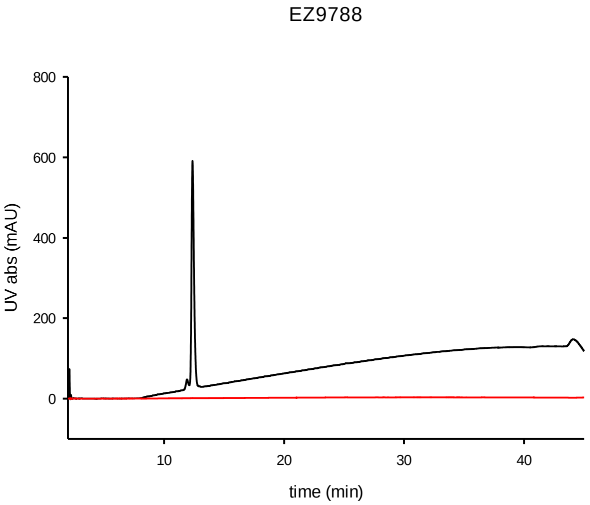

HMGN1_unmodN (Yield after ligation, desulfurisation and purification: ~ 59%, 3.2 mg for unlabelled; ~11%, 0.6 mg for ^{15}N-labelled)

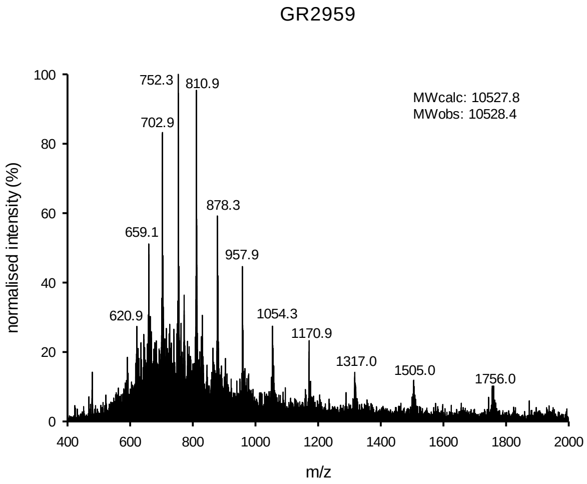

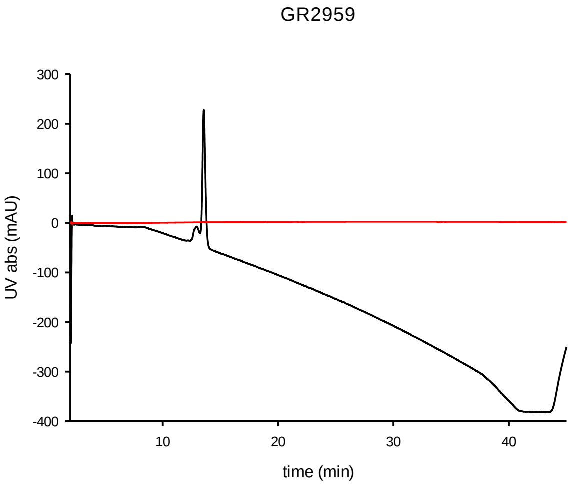

HMGN1_acK2 (Yield after ligation, desulfurisation and purification: ~ 86%, 4.7 mg for unlabelled; ~9%, 0.5 mg for ^{15}N-labelled)

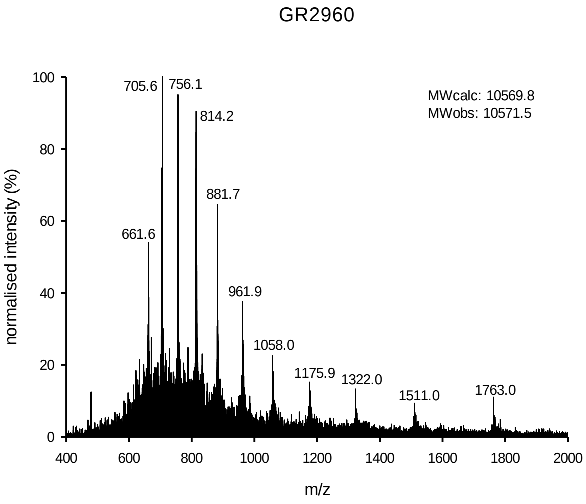

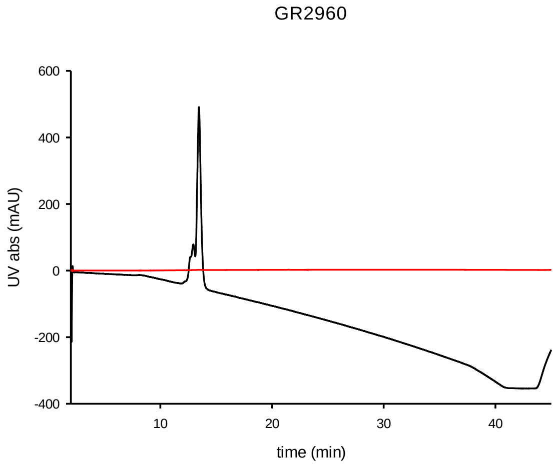

HMGN1_pS6 (Yield after ligation, desulfurisation and purification: ~ 60%, 3.3 mg for unlabelled; ~9%, 0.5 mg for ^{15}N-labelled)

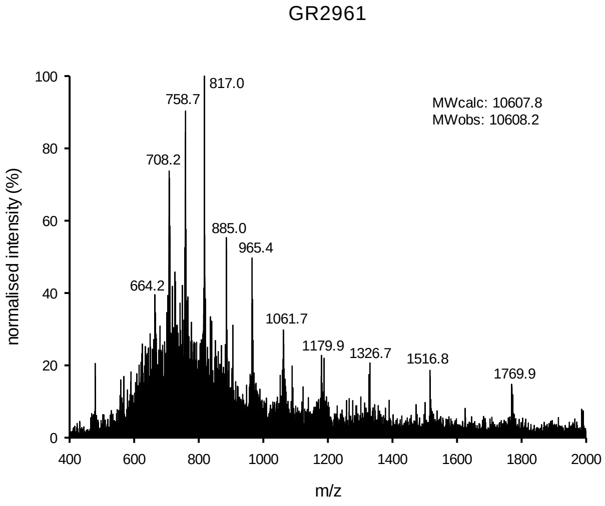

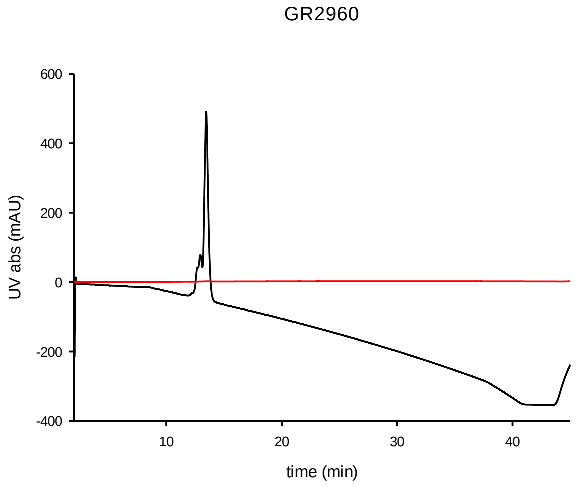

HMGN1_acK2_pS6 (Yield after ligation, desulfurisation and purification: ~ 33%, 1.8 mg for unlabelled; ~11%, 0.6 mg for ^{15}N-labelled)

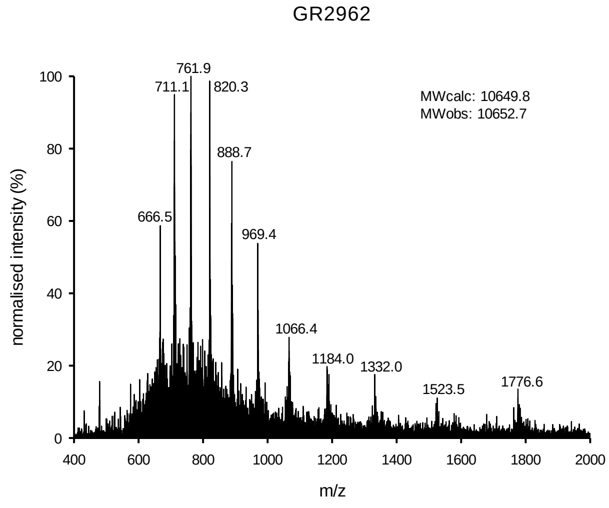

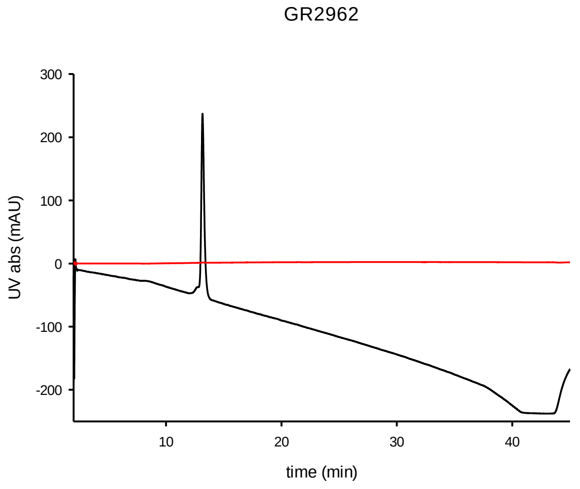

HMGN1_unmod_C (Yield after ligation, desulfurisation and purification: ~ 21%, 1.0 mg for unlabelled; ~42%, 0.3 mg for ^{15}N-labelled)

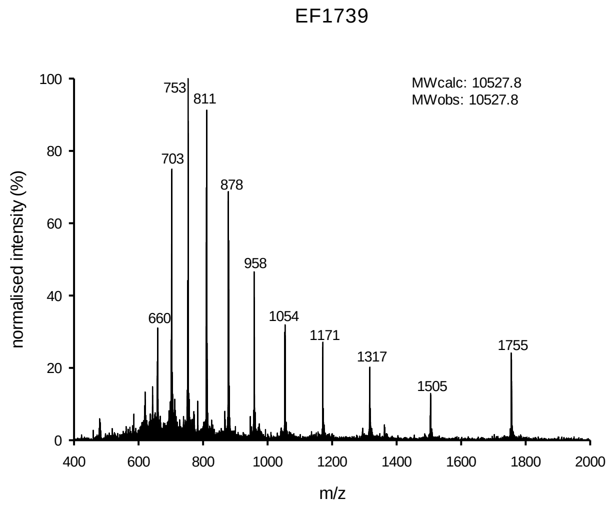

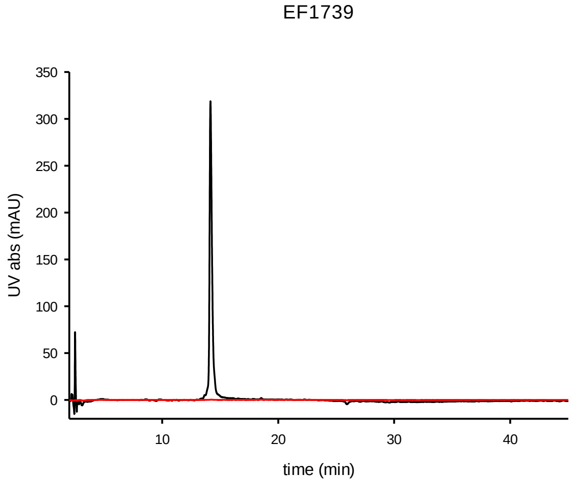

HMGN1_pS85 (Yield after ligation, desulfurisation and purification: ~ 12%, 0.6 mg for unlabelled)

HM0002

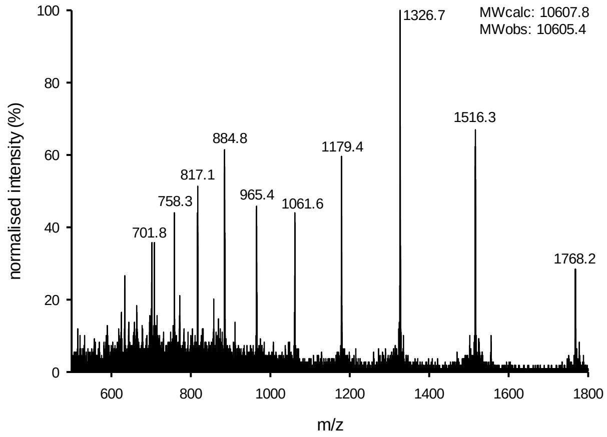

HM0002

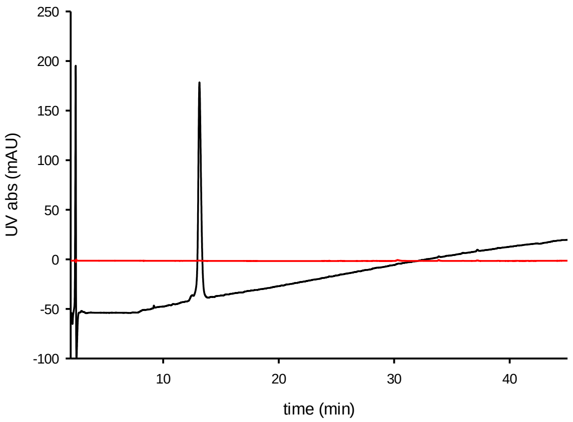

HMGN1_pS88 (Yield after ligation, desulfurisation and purification: ~ 21%, 1.0 mg for unlabelled; ~13%, 0.4 mg for ^{15}N-labelled)

EF1740

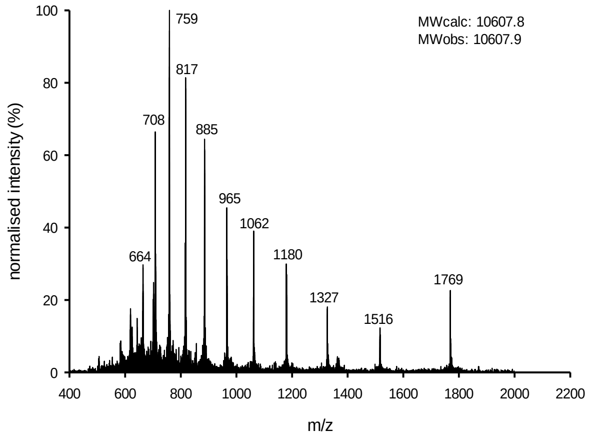

EF1740

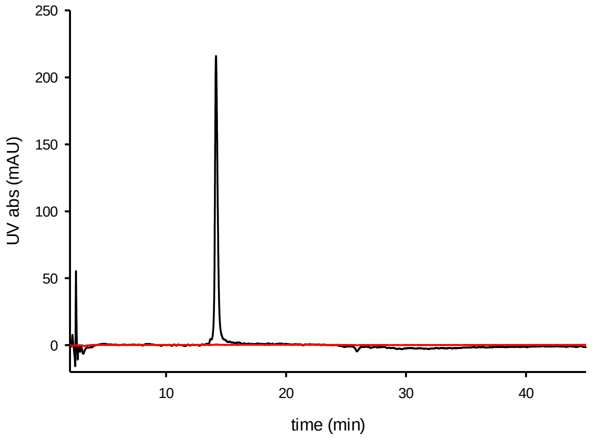

HMGN1_pS85,88,98 (Yield after ligation, desulfurisation and purification: ~ 29%, 1.4 mg for unlabelled; ~11%, 0.3 mg for ^{15}N-labelled)

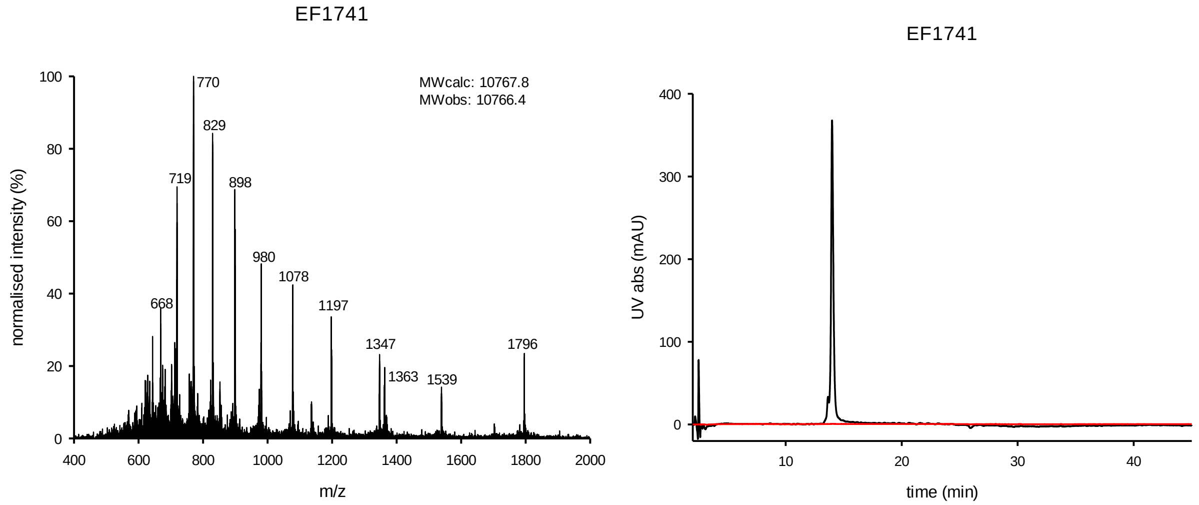

HMGN1_1-64 (Yield from expression, purification, intein cleavage and HPLC purification: 5.7 mg/L culture for unlabelled; 0.6 mg/L of rich medium, 250 mL minimal medium for ^{15}N-labelled ~ 16%, 0.5 mg after hydrolysis and repurification, unlabelled)

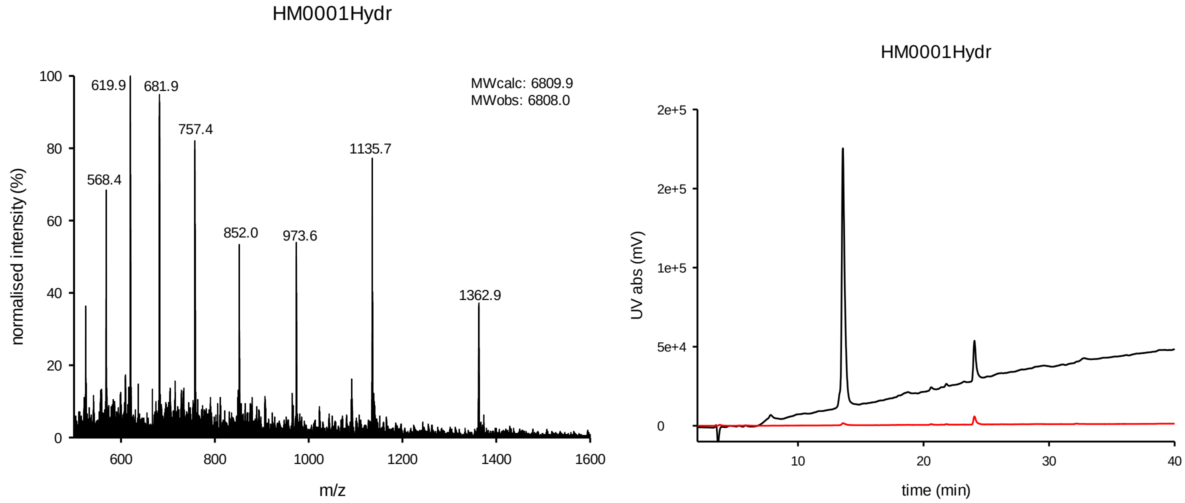

HMGN1_11-99 (Yield from expression, purification, TEV cleavage and HPLC purification: 6.7 mg/L culture for unlabelled; 3.8 mg/L of rich medium, 250 mL minimal medium for ^{15}N-labelled ~ 65%, 5.4 mg for desulfurisation and repurification, unlabelled)

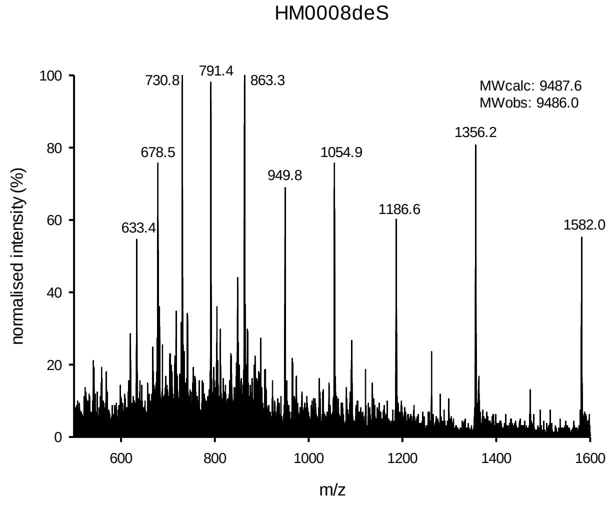

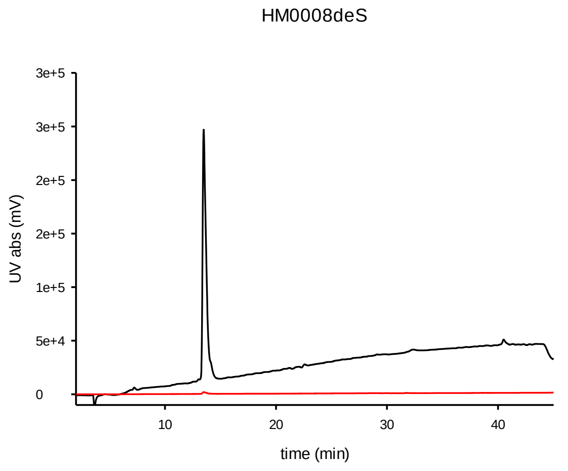

HMGN1_65-99 (Yield: 22% after HPLC purification, relative to calculated yield from synthesis scale of 0.05 mmol)

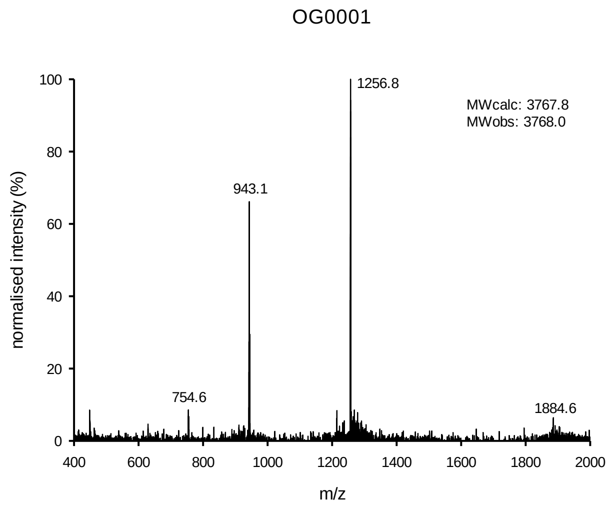

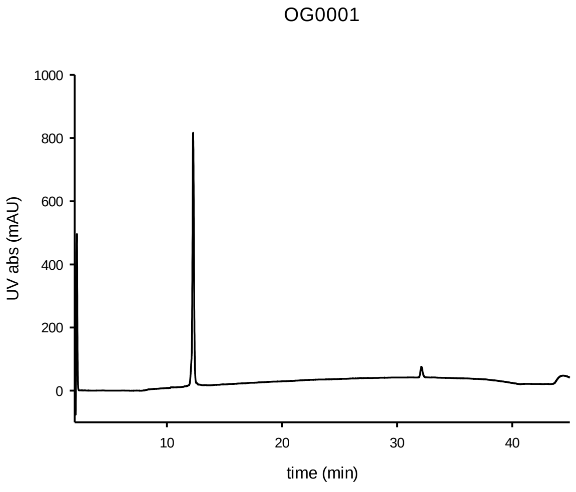

HMGN1_65-99_pS88 (Yield: 28% after HPLC purification, relative to calculated yield from synthesis scale of 0.1 mmol)

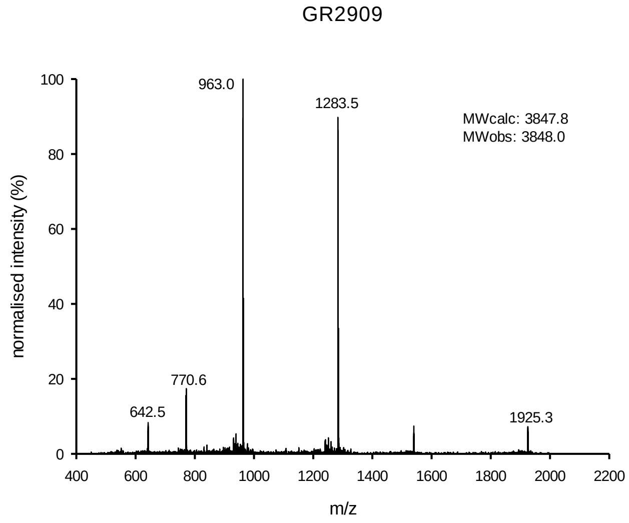

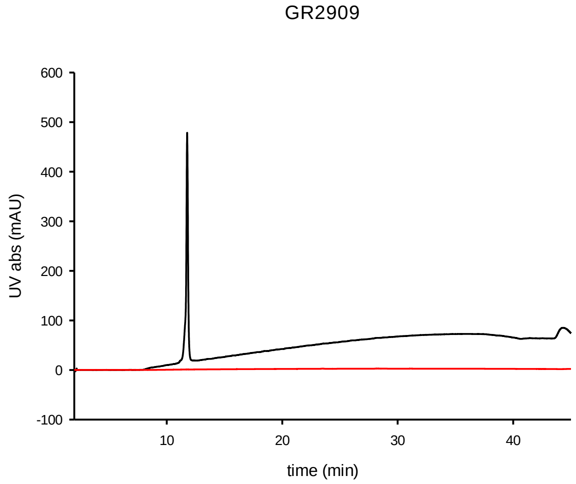

HMGN1_65-99_pS85 (Yield: 32% after HPLC purification, relative to calculated yield from synthesis scale of 0.1 mmol)

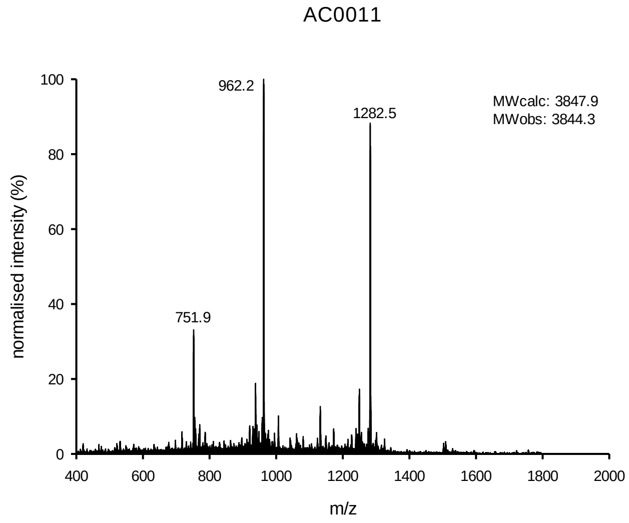

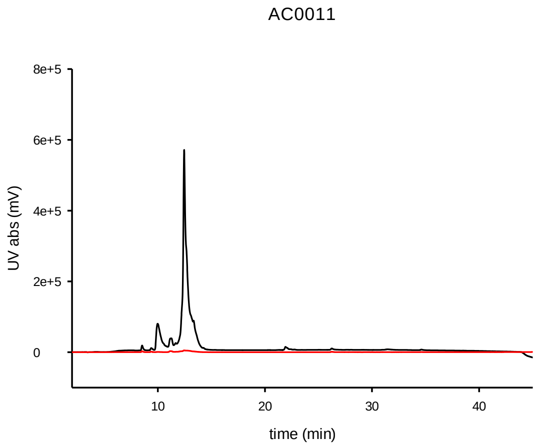

HMGN1_65-99_pS85,88,98 (Yield: 16% after HPLC purification, relative to calculated yield from synthesis scale of 0.1 mmol)

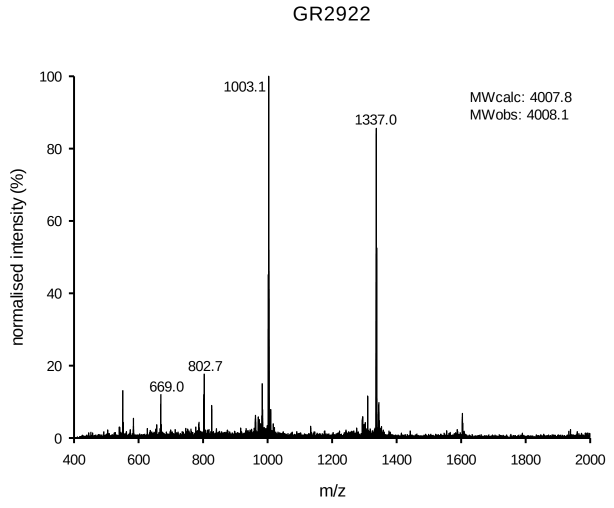

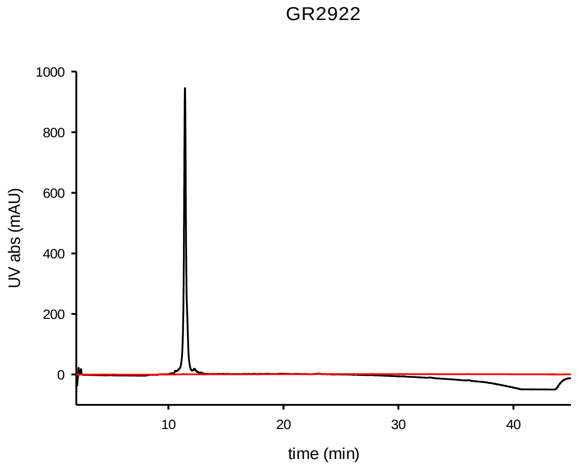

HMGN1_1-26 (Yield: 58% after HPLC purification, relative to calculated yield from synthesis scale of 0.05 mmol)

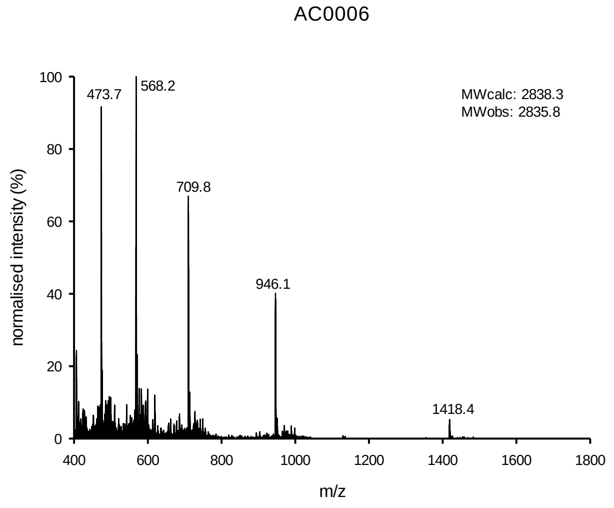

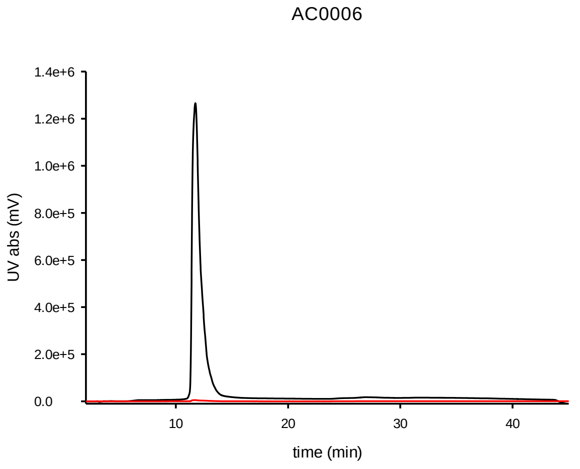

HMGN1_1-26_acK2,pS6 (Yield: 55% after HPLC purification, relative to calculated yield from synthesis scale of 0.05 mmol)

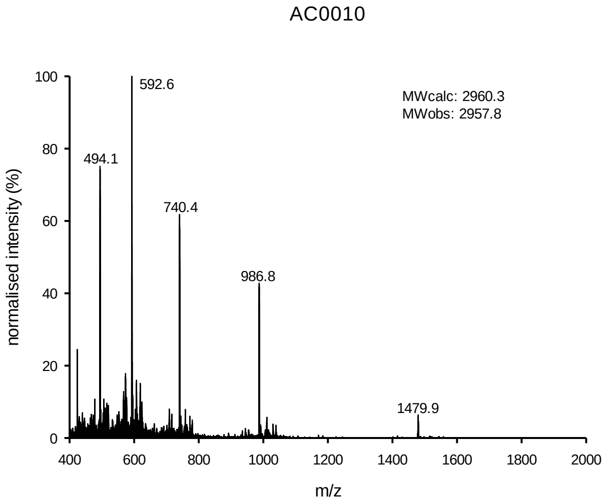

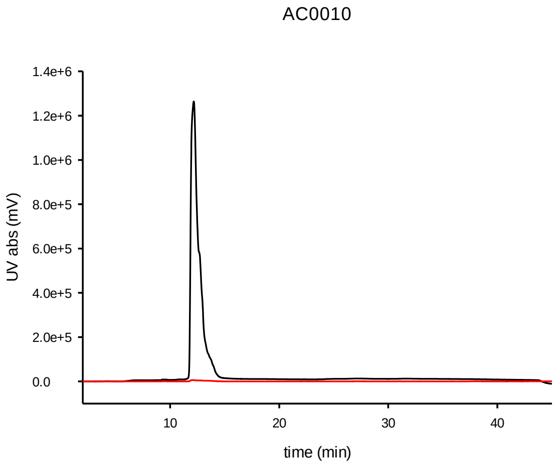

4. **Chemical structures, mass spectra and analytical HPLC data for synthetic N-terminal HMGN1 hydrazide segments for ligation.**

HMGN1_1-10-hydrazide (Yield: 54 % after HPLC purification, relative to calculated yield from synthesis scale of 0.05 mmol)

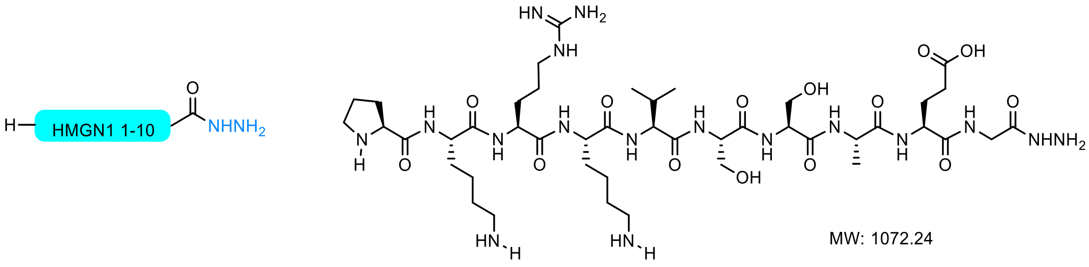

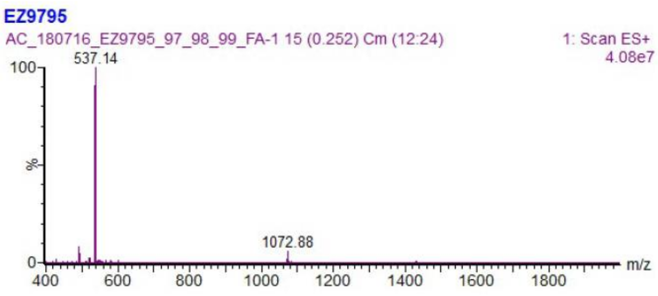

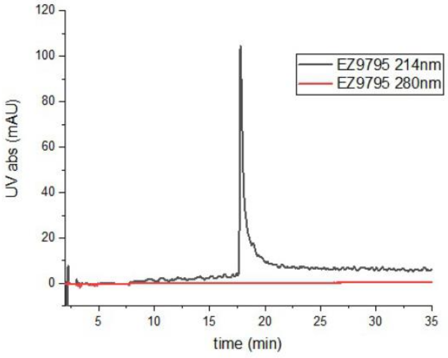

HMGN1_1-10_acK2-hydrazide (Yield: 37% after HPLC purification, relative to calculated yield from synthesis scale of 0.05 mmol)

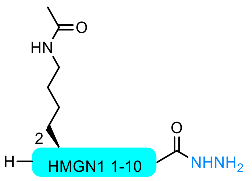

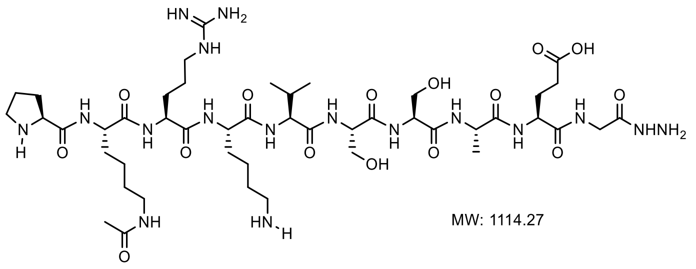

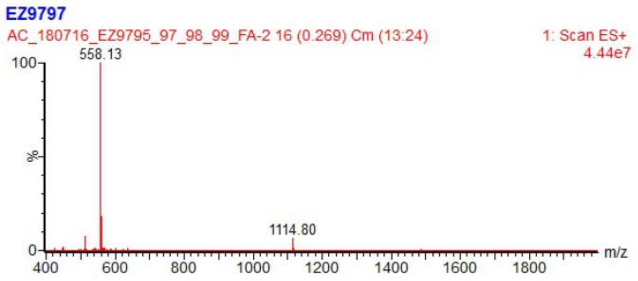

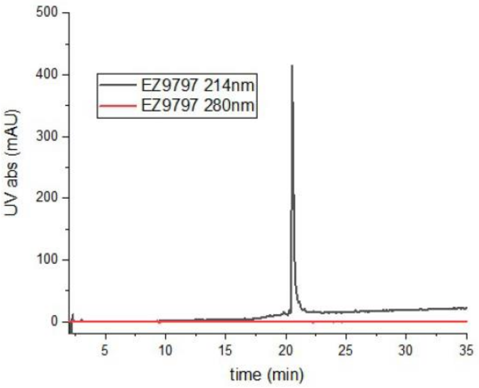

HMGN1_1-10_pS6-hydrazide (Yield: 30% after HPLC purification, relative to calculated yield from synthesis scale of 0.05 mmol)

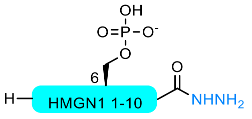

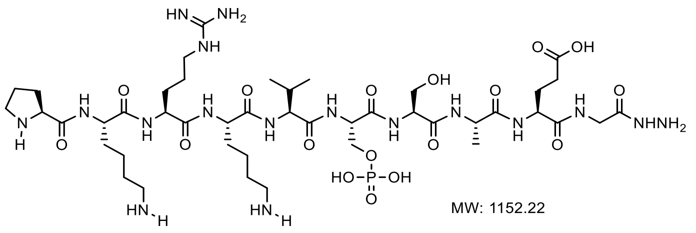

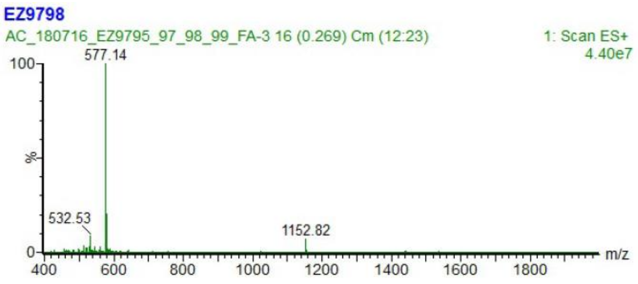

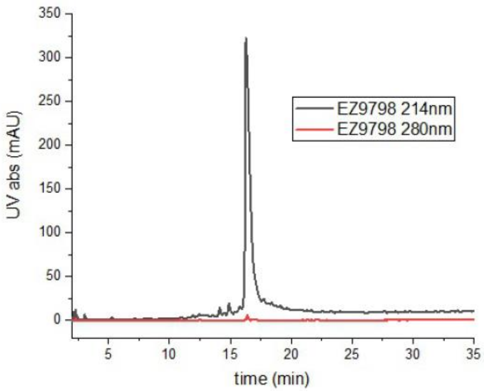

HMGN1_1-10_acK2,pS6-hydrazide (Yield: 17% after HPLC purification, relative to calculated yield from synthesis scale of 0.05 mmol)

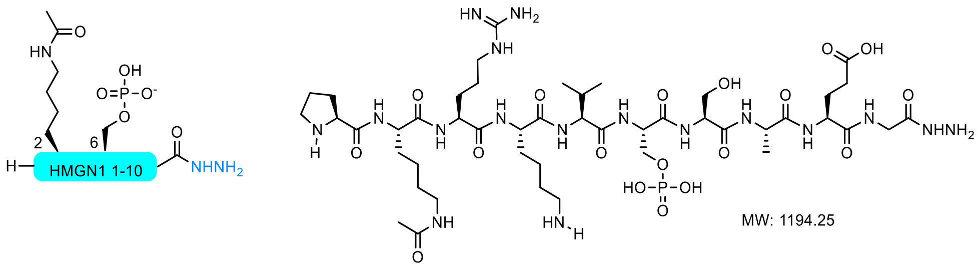

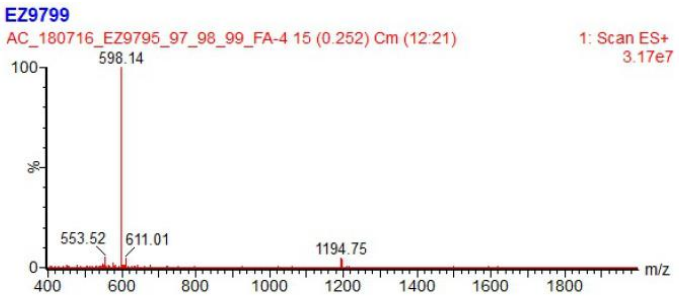

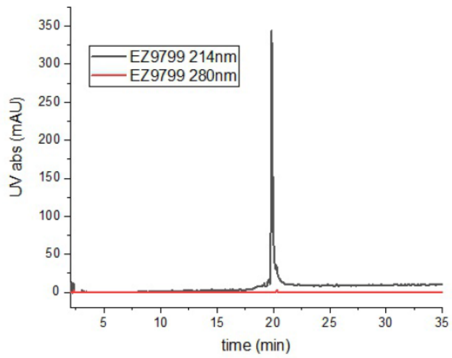

5. **SDS PAGE gels**

5.1 SDS PAGE gel (15%): Ligation and desulfurisation of HMGN1 bearing N-terminal PTMs

Lanes: 1 – marker; 2 – HMGN1_11-99_A11C before ligation; 3 – HMGN1_unmod_N ligation 4 h; 4 – HMGN1_acK2 ligation 4 h; 5 – HMGN1_pS6 ligation 4 h; 6 – HMGN1_acK2,pS6 ligation 4 h; 7 – HMGN1_unmod_N desulfurised; 8 – HMGN1_acK2 desulfurised; 9 – HMGN1_pS6 desulfurised; 10 - HMGN1_acK2,pS6 desulfurised.

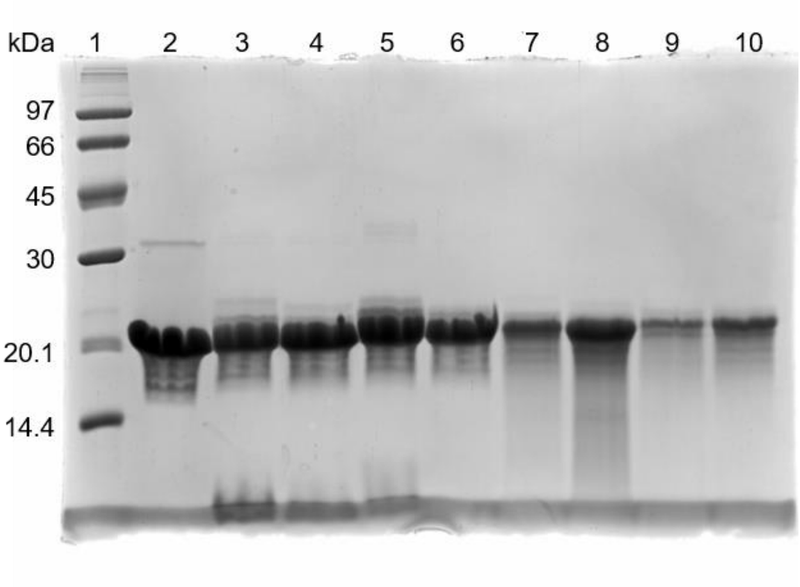

5.2 SDS PAGE gel (15%): Intein cleavage of HMGN1_1-65_MxeHis7CBD.

Lanes: 1 – marker; 2 – HMGN1_1-64_MxeHis7_CBD; 3 – intein cleavage with 250 mM MesNa 0 h; 4 – intein cleavage with 250 mM MesNa 16 h. HMGN1_1-64_MxeHis7_CBD = 35.9 kDa; Cleaved Mxe intein = 29.1 kDa; HMGN1_1-64 thioester = 6.9 kDa.

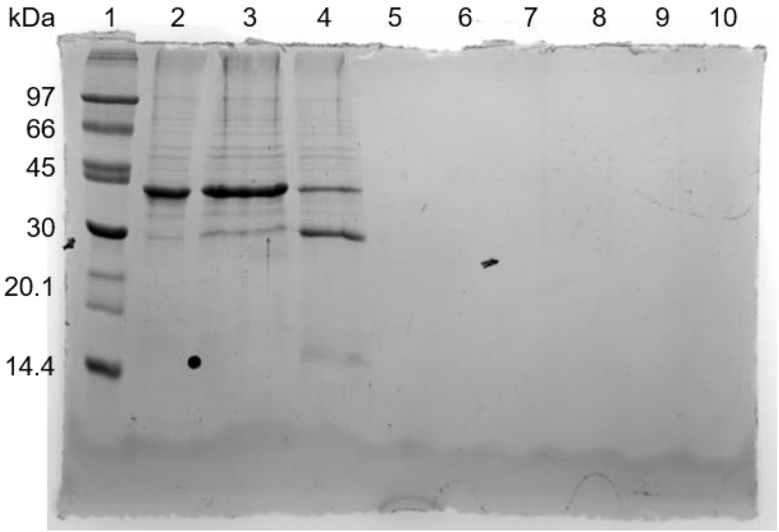

5.3 SDS PAGE gel (15%): Ligation of HMGN1 bearing C-terminal PTMs.

Lanes: 1 – marker; 2 – HMGN1_unmod_C ligation 0 h; 3 – HMGN1_pS88 ligation 0 h; 4 – HMGN1_pS85,88,98 ligation 0 h; 5 – HMGN1_unmod_C ligation 16 h; 6 – HMGN1_pS88 ligation 16 h; 7 – HMGN1_pS85,88,98 ligation 16 h.

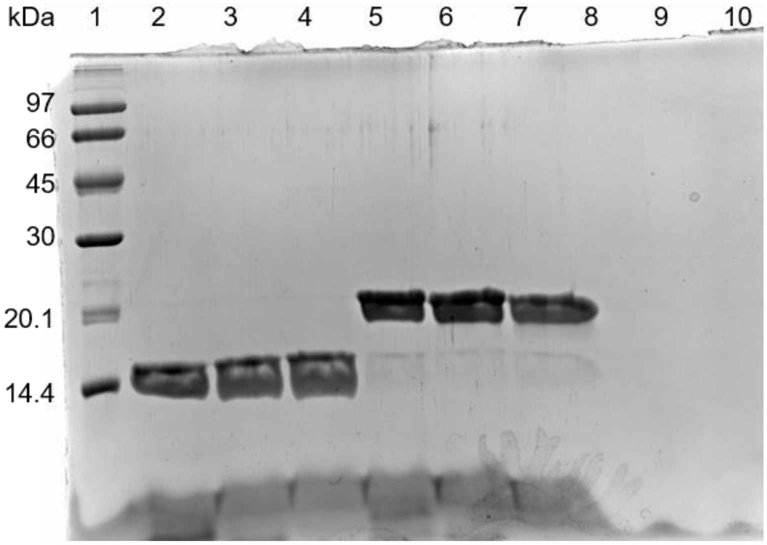

6. Full NMR spectra

6.1 ^{1}H-^{15}N HSQC NMR spectrum of uniformly labelled, unmodified HMGN1 with residue assignments labelled using single-letter amino acid codes. Four low intensity peaks (marked with *) could not be assigned and are proposed to arise from minor conformations of residues around 75-79. The proposed residue types are a* = Gly, b* = Gln/Glu, c* = Asp, d* = Ala and they are found in the 65-99 segment as they are not observed in the variants were only residues 1-64 are labelled.

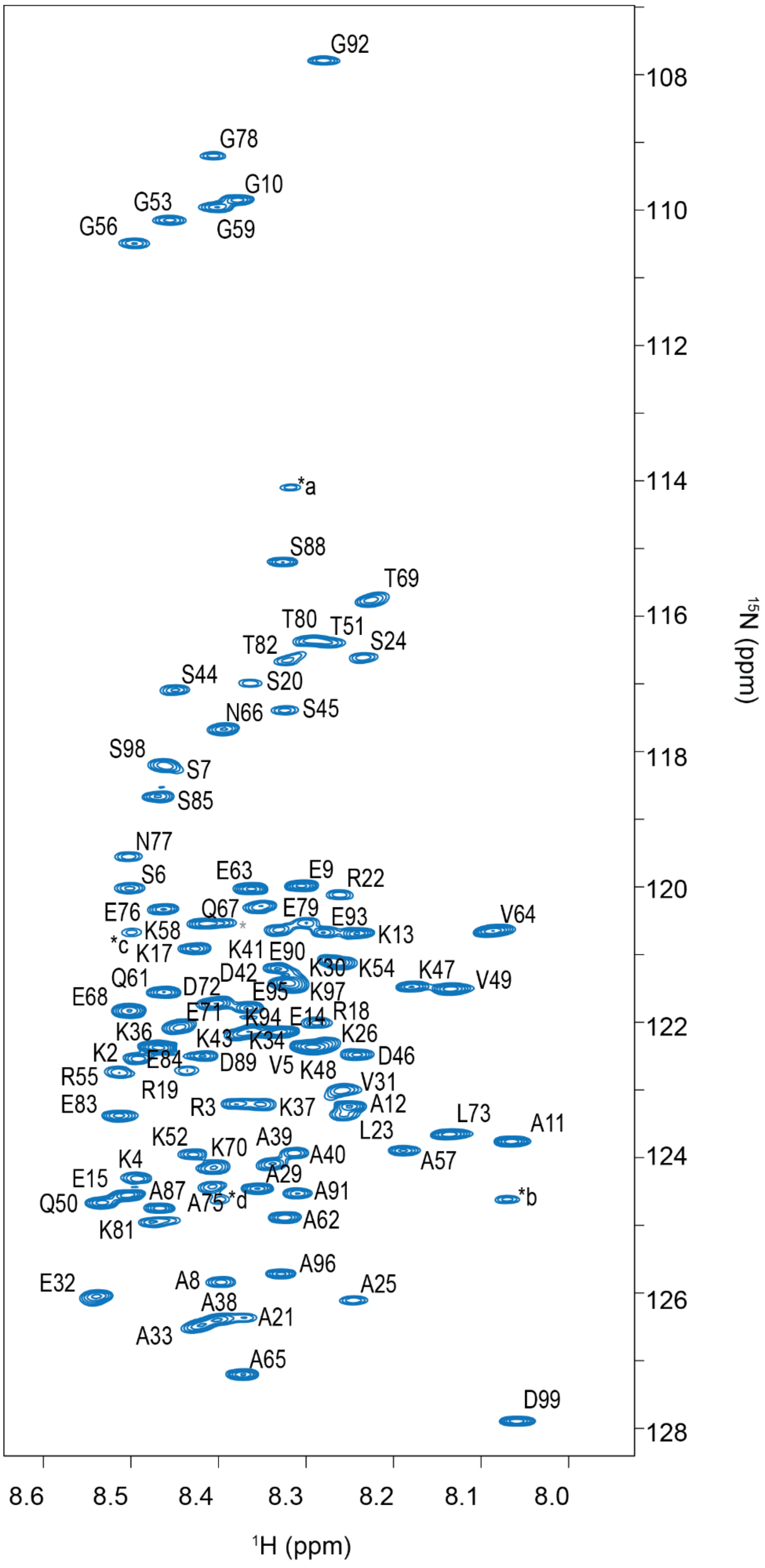

G92
G78
G10
G53
G56
G59
*a
S88
T69
T80
T51
S24
T82
S44
S20
N66
S45
S98
S7
S85
N77
E63
E9
R22
S6
E76
Q67
E79
E93
K58
K13
V64
*c
K17
K41
E90
K30
K54
K47
V49
Q61
D42
D72
E95
K97
E68
E71
K94
E14
R18
K36
K43
K34
K26
K2
E84
D89
V5
K48
D46
R55
R19
V31
E83
R3
K37
A12
L73
L23
A11
K52
K70
A39
A40
A57
K4
A29
E15
A87
A91
*b
Q50
A75
*d
A62
K81
A96
E32
A8
A25
A38
A21
A33
A65
D99
108
110
112
114
116
118
120
122
124
126
128
¹⁵N (ppm)
8.6
8.5
8.4
8.3
8.2
8.1
8.0
¹H (ppm)

6.2 ^{15}N-HSQC spectra of HMGN1_1-99_15N13C (black), HMGN1_unmodN_15N (pink), HMGN1_acK2_15N (blue), HMGN1_pS6_15N (purple), and HMGN1_acK2,pS6_15N (green). Section shown in Figure 3b marked with a dashed box. Below is the section shown in Figure 3b with all residue labels.

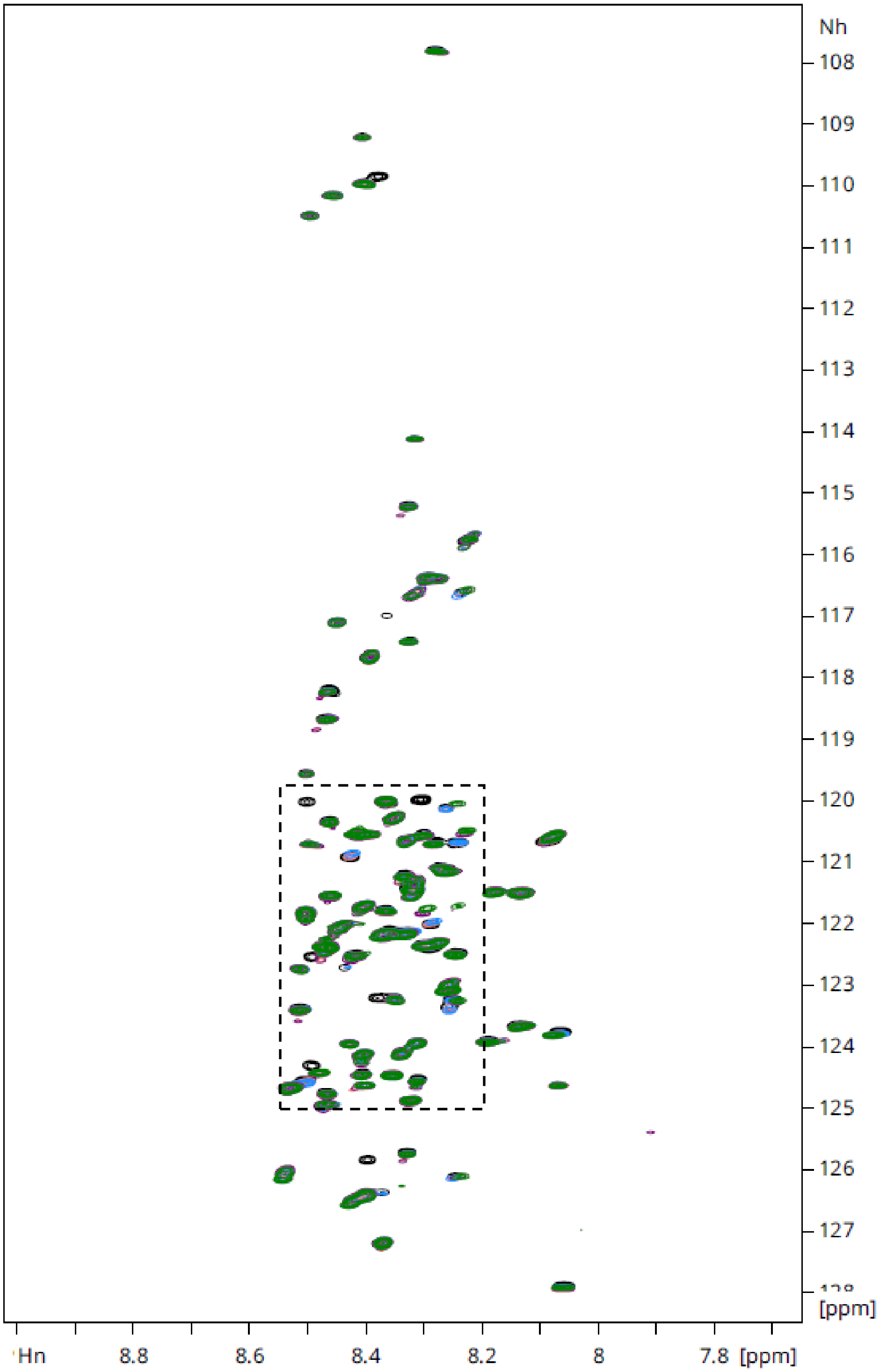

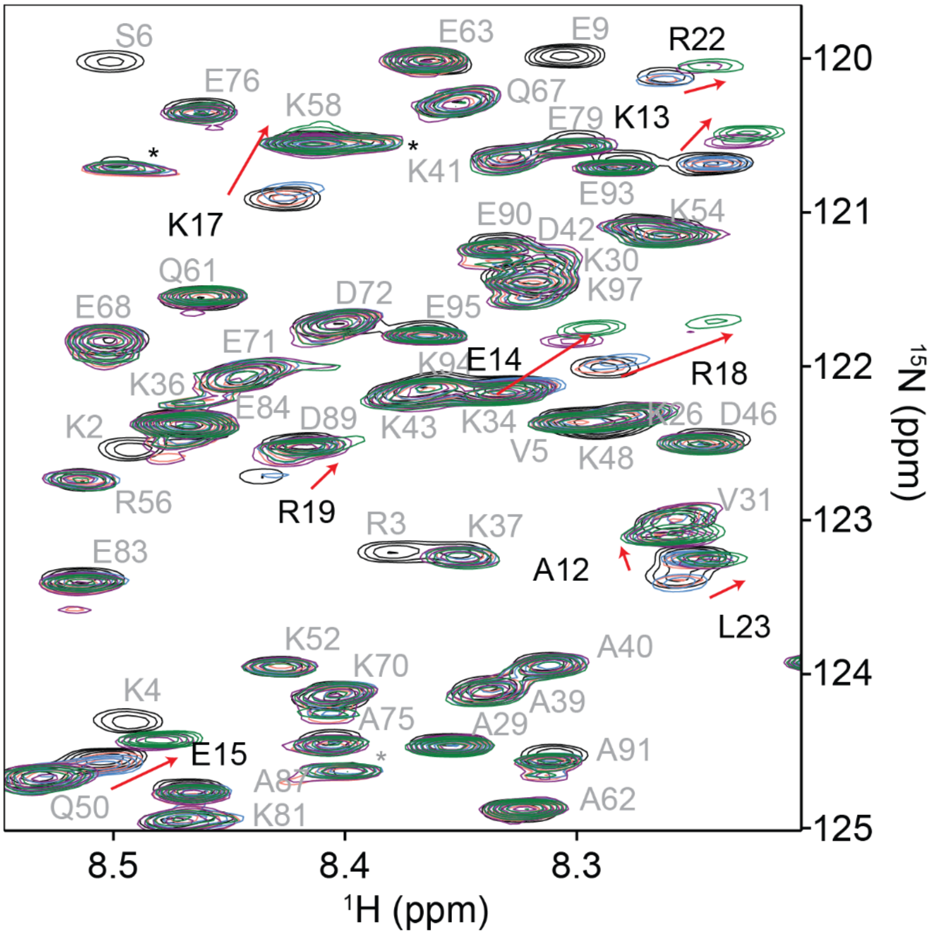

6.3 ^{15}N-HSQC spectra of HMGN1_1-99_15N13C (black), HMGN1_unmodN_15N (pink), HMGN1_acK2_15N (blue), HMGN1_pS6_15N (purple), and HMGN1_acK2,pS6_15N (green) acquired in 25 mM NaCl/25 mM KCl, corresponding to the salt concentrations in the electrophoretic gel mobility assay. Residues are labelled as in Figure 3b.

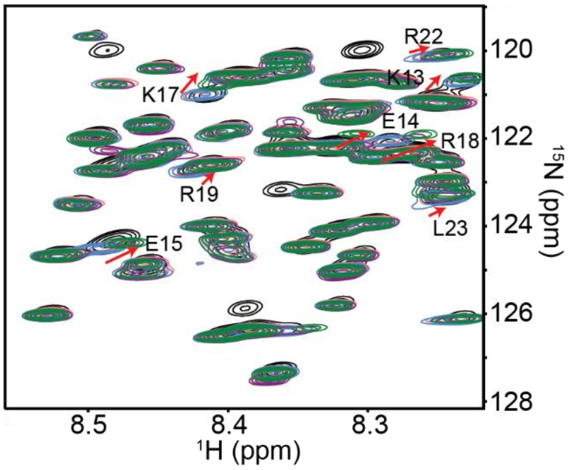

6.4 ^{15}N-HSQC spectra of HMGN1_1-26 (black) and HMGN1_1-26_acK2,pS6 (blue). Section shown in Figure 3c marked with a dashed box.

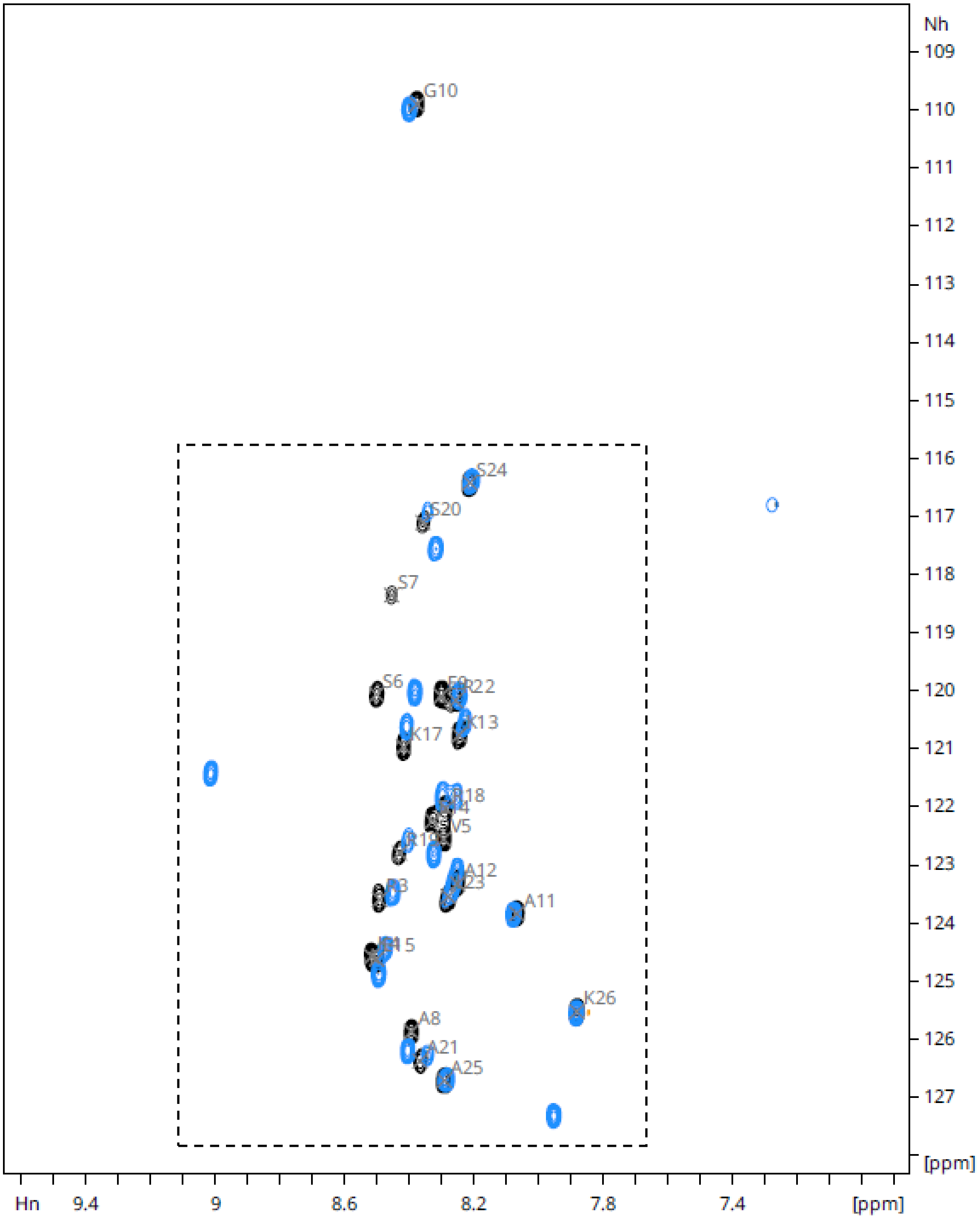

6.5 ^{15}N-HSQC spectra of HMGN1_1-99_15N13C (black), HMGN1_unmodC_15N (green), HMGN1_pS88_15N (purple), and HMGN1_pS85,88,98_15N (coral). Section shown in Figure 4b marked with a dashed box. Below is the section shown in Figure 4b with all residue labels.

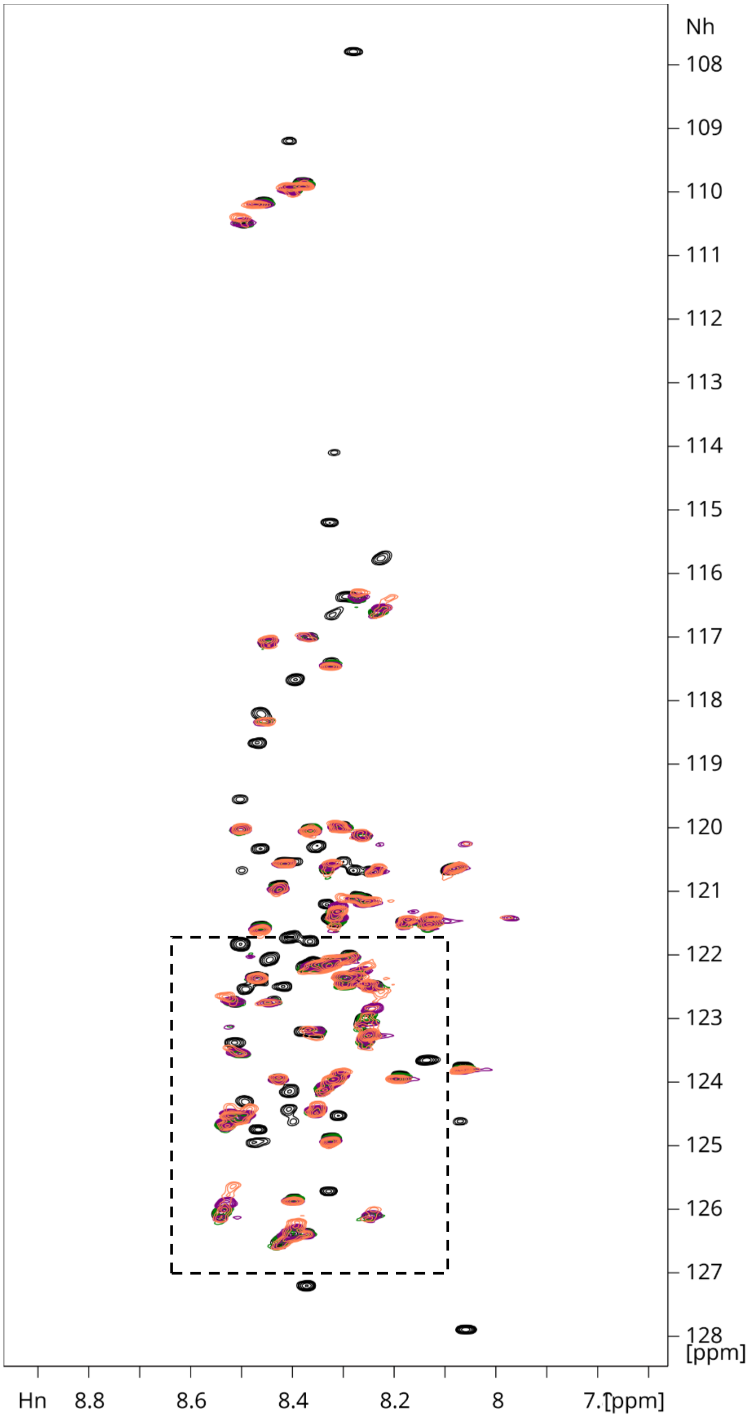

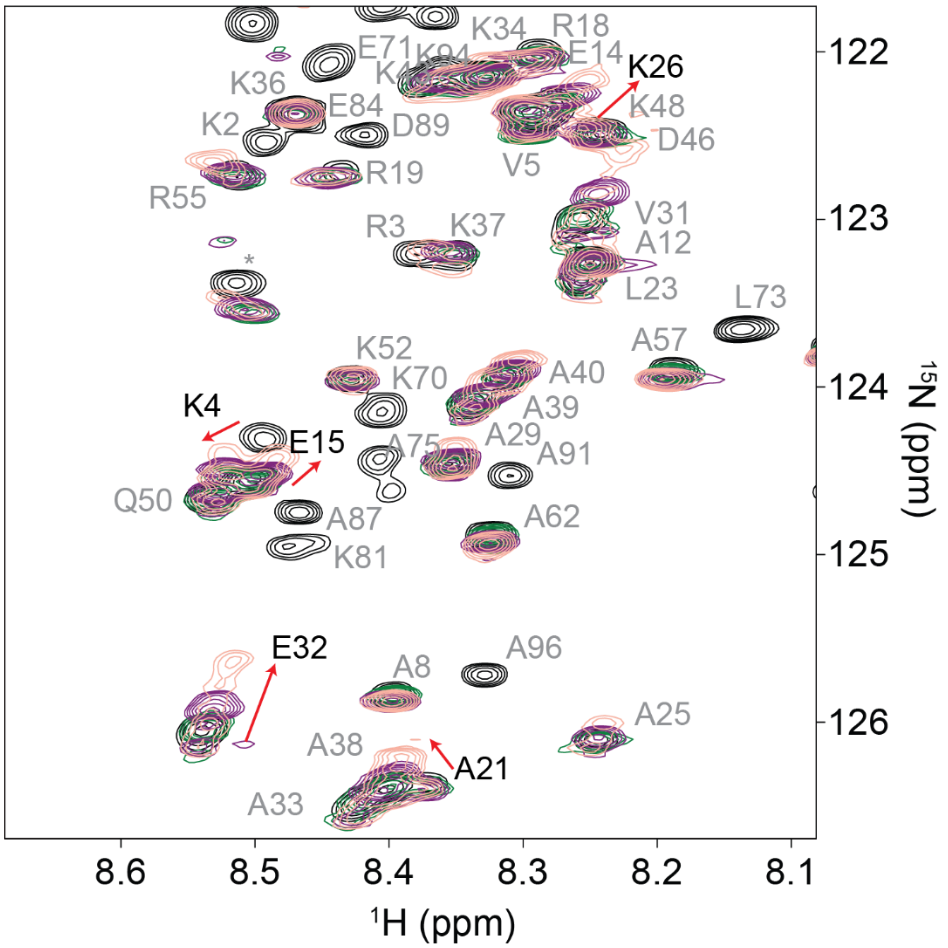

K34
R18
E71
K94
E14
K26
K36
E84
K48
K2
D89
D46
V5
R19
R55
V31
R3
K37
A12
*
L23
L73
A57
K52
K70
A40
A39
K4
E15
A75
A29
A91
Q50
A87
A62
K81
E32
A96
A8
A25
A38
A21
A33
122
123
124
125
126
^{15}N (ppm)
8.6
8.5
8.4
8.3
8.2
8.1
^{1}H (ppm)

6.6 ^{15}N-HSQC spectra of HMGN1_65-99_A65C (black) and HMGN1_65-99_pS85,88,98_A65C (blue). Section shown in Figure 4c marked with a dashed box. Numbering is shown as (n+1) relative to the sequence numbering in Figure 4c.

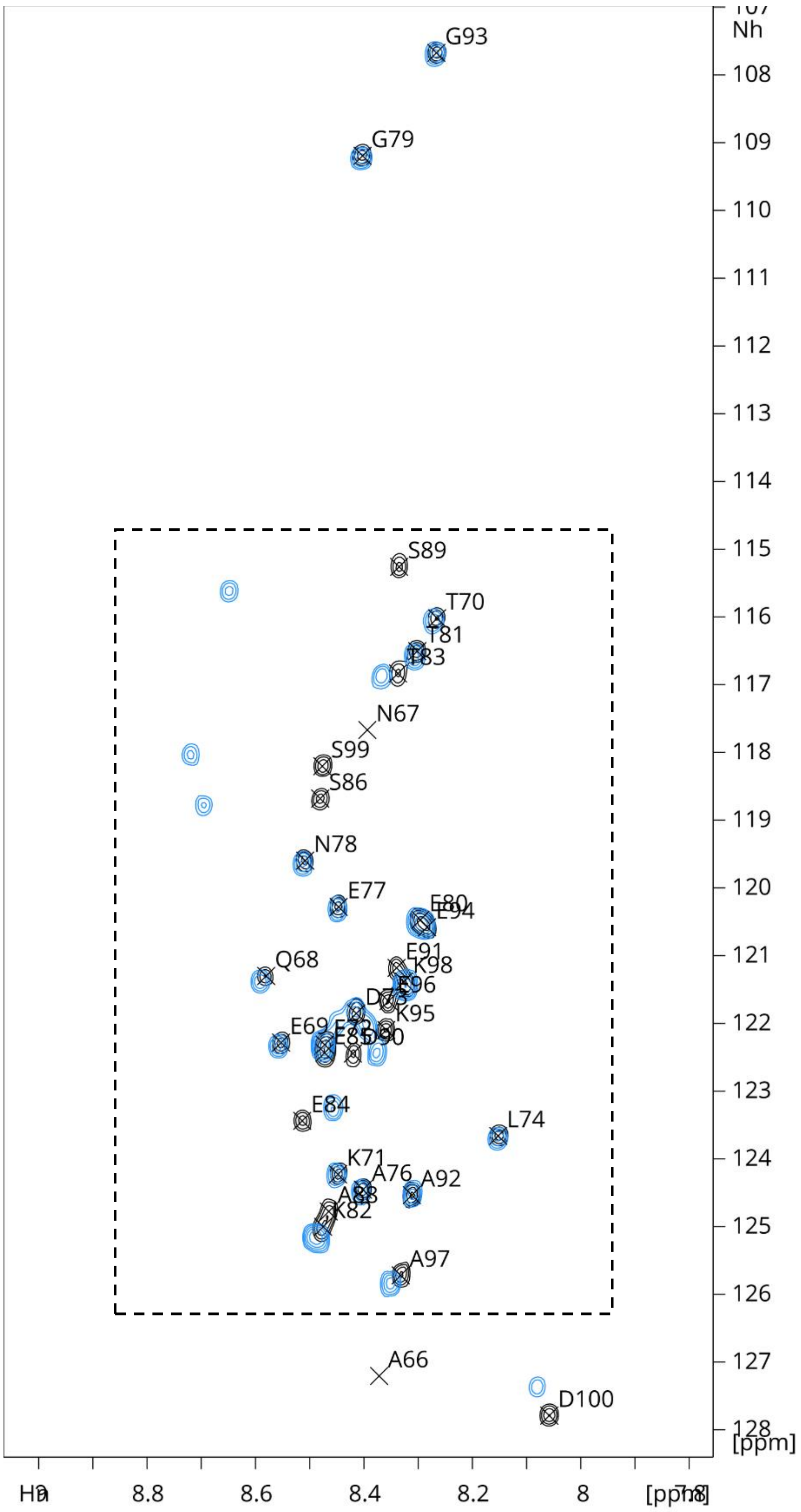

6.7 ^{15}N-HSQC spectra of HMGN1_65-99_A65C (black), HMGN1_65-99_pS88_A65C (turquoise), HMGN1_65-99_pS85_A65C (red) and HMGN1_65-99_pS85,88,98_A65C (blue). Numbering is shown as (n+1) relative to the sequence numbering in Figure 4c.

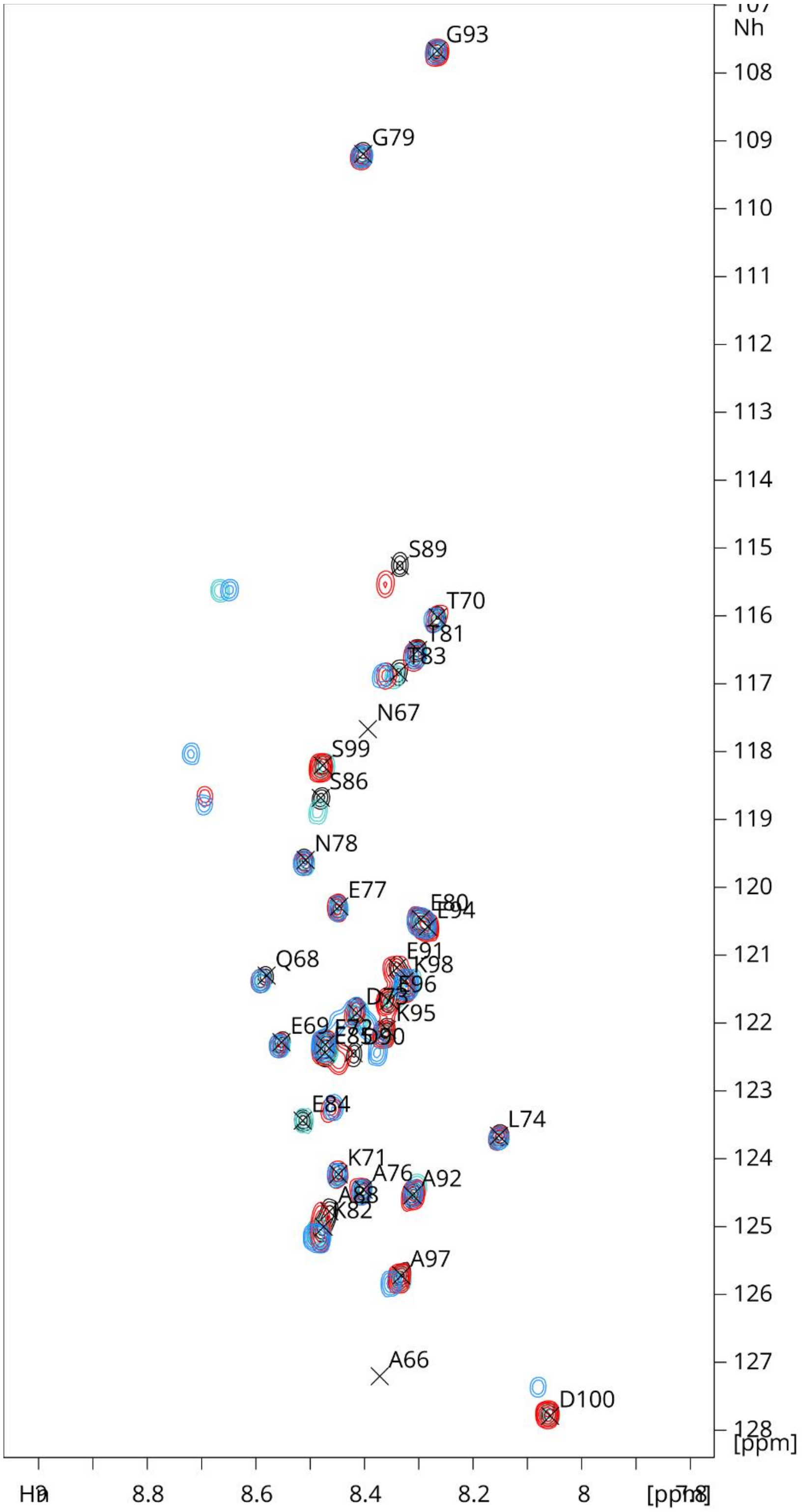

6.8 Structures of short model peptides containing Ser-Pro and pSer-Pro residues in their *cis*- and *trans*-conformations. ^{15}N-HSQC spectra of Ser-Pro containing peptide (black) and pSer-Pro containing peptide (blue) showing shifts upon phosphorylation. Minor conformations corresponding to the *cis*-Pro population are visible as low intensity peaks.

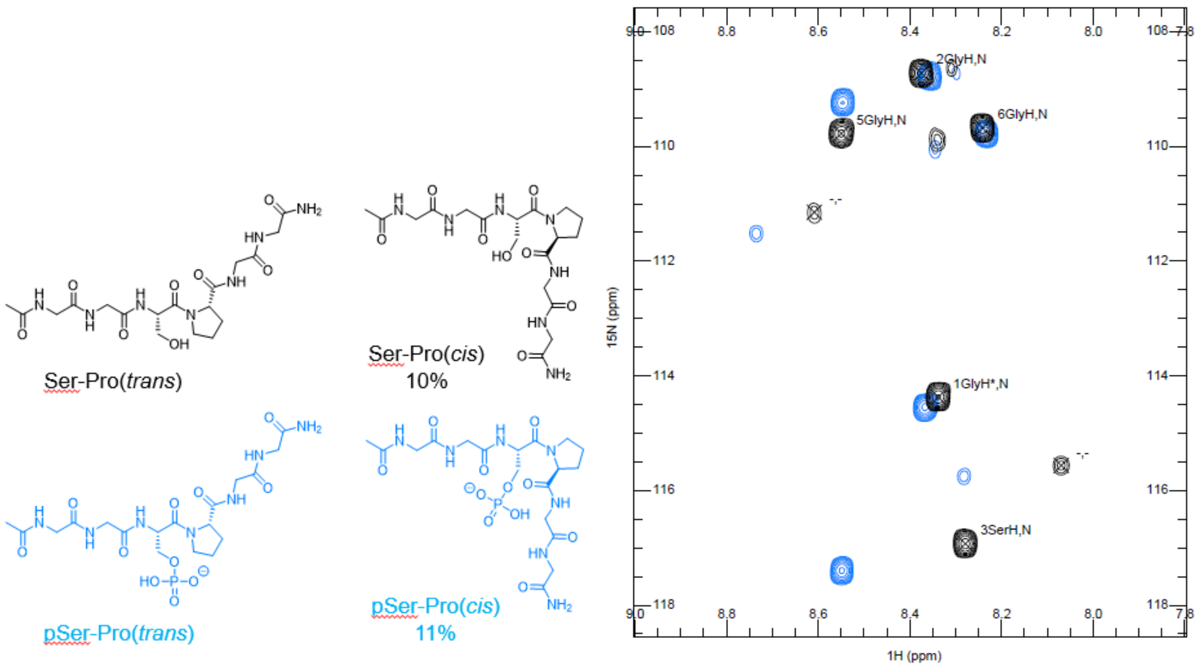

7 Heteronuclear ^1H-^{15}N NOE ratios

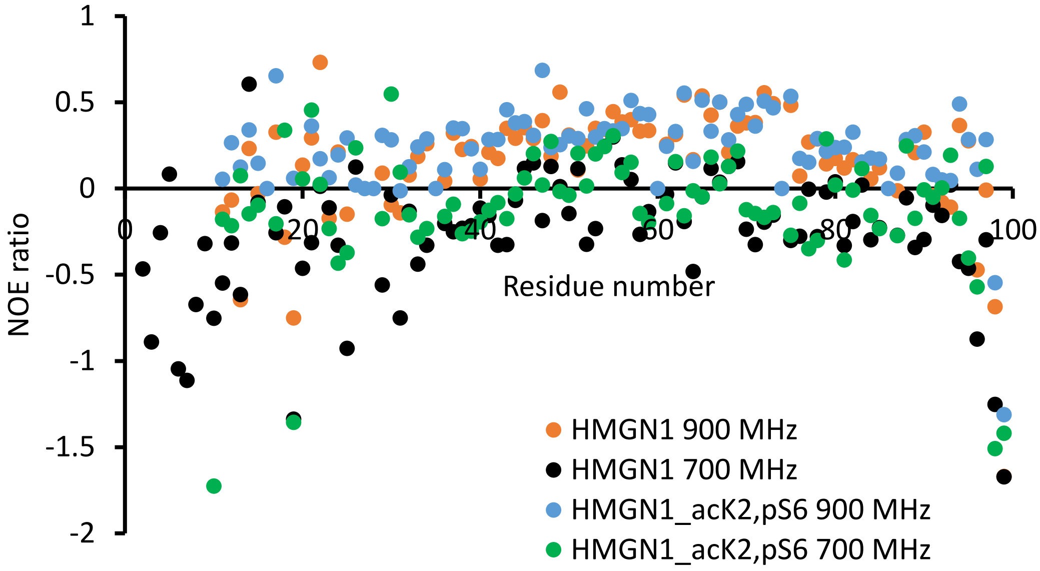

Figure S7.1. ^1H-^{15}N heteronuclear NOE ratios for unmodified HMGN1 (HMGN1_S0_15N) and HMGN1_acK2,pS6_15N at 700 MHz and 900 MHz.

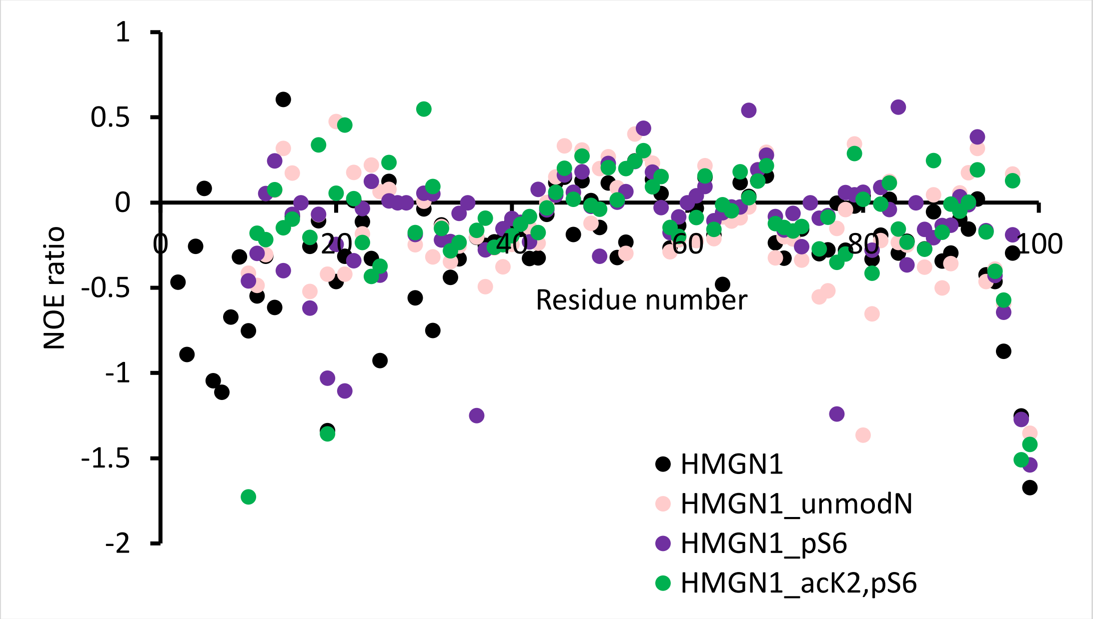

Figure S7.2. ^1H-^{15}N heteronuclear NOE ratios for unmodified HMGN1 (HMGN1_S0_15N), HMGN1_unmodN_15N, HMGN1_pS6_15N and HMGN1_acK2,pS6_15N at 700 MHz.

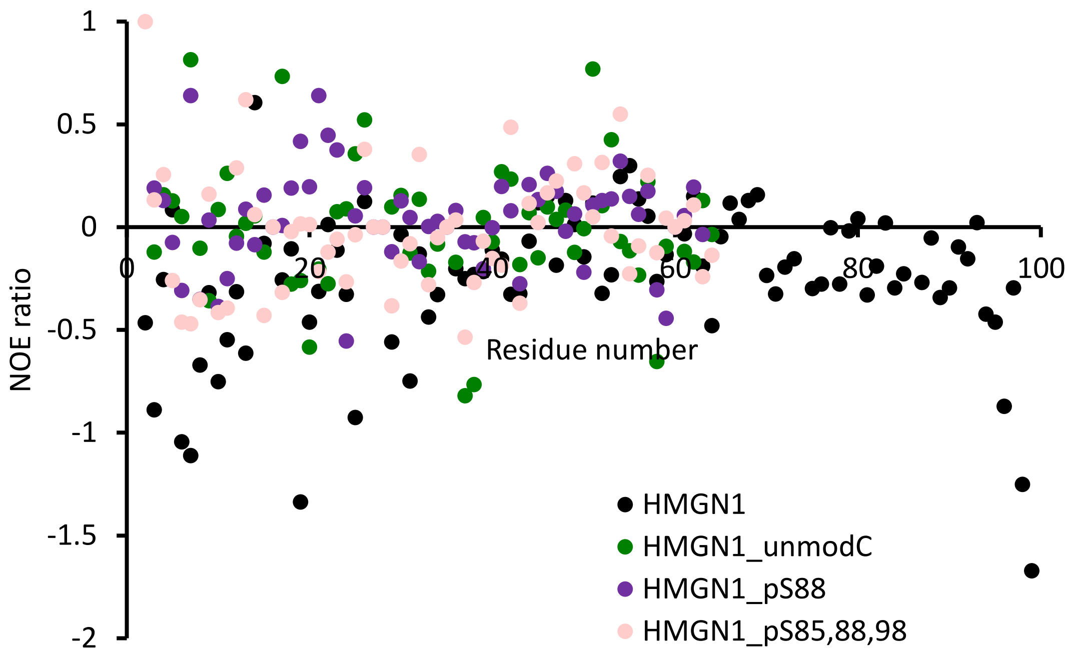

Figure S7.3. ^{1}H-^{15}N heteronuclear NOE ratios for unmodified HMGN1 (HMGN1_S0_15N), HMGN1_unmodC_15N, HMGN1_pS88_15N and HMGN1_acK2,pS6_15N at 700 MHz.

8.1 Backbone chemical shifts (ppm) of unmodified HMGN1

Residue	AA	NH	HN	CA	CB	CO
0	S					
1	P					
2	K	122.5	8.49	56.4	33.1	176.6
3	R	123.2	8.38	56.0	31.2	176.0
4	K	124.3	8.49	56.4	33.1	176.5
5	V	122.4	8.30	62.1	33.0	176.2
6	S	120.0	8.50	58.2	64.1	174.7
7	S	118.3	8.46	58.5	63.9	174.5
8	A	125.8	8.40	52.9	19.1	178.0
9	E	120.0	8.31	57.0	30.2	177.3
10	G	109.8	8.38	45.4		174.0
11	A	123.8	8.07	52.5	19.3	177.7
12	A	123.2	8.25	52.5	19.1	177.8
13	K	120.7	8.24	56.3	33.2	176.5
14	E	122.1	8.33	56.1	30.6	176.2
15	E	124.5	8.51	54.7	29.7	174.7
16	P					
17	K	120.9	8.43	56.6	32.8	177.0
18	R	122.0	8.29	56.3	30.8	176.5
19	R	122.7	8.44	56.5	30.8	176.5
20	S	117.0	8.37	58.5	63.8	174.5
21	A	126.4	8.37	52.8	19.3	177.8
22	R	120.1	8.26	56.4	30.7	176.5
23	L	123.4	8.26	55.2	42.3	177.4
24	S	116.6	8.24	58.2	63.9	174.0
25	A	126.1	8.25	52.3	19.4	177.3
26	K	122.3	8.28	54.1	32.5	174.1
27	P					
28	P					
29	A	124.4	8.36	52.3	19.3	177.7
30	K	121.4	8.32	56.2	33.2	176.4
31	V	123.0	8.26	62.2	33.0	176.0
32	E	126.1	8.54	56.1	30.6	175.8
33	A	126.5	8.42	52.3	19.3	177.4
34	K	122.1	8.34	54.1	32.5	174.5
35	P					
36	K	122.4	8.48	56.4	33.3	176.7
37	K	123.2	8.35	56.2	33.2	176.1
38	A	126.4	8.40	52.3	19.3	177.3

39	A	124.1	8.34	52.2	19.4	177.5
40	A	123.9	8.32	52.5	19.3	177.9
41	K	120.6	8.33	56.4	33.1	176.4
42	D	121.4	8.32	54.3	41.4	176.4
43	K	122.2	8.38	56.5	32.9	177.1
44	S	117.1	8.45	59.1	63.8	175.0
45	S	117.4	8.33	58.7	63.8	174.4
46	D	122.5	8.25	54.6	41.1	176.3
47	K	121.5	8.18	56.4	32.9	176.7
48	K	122.4	8.29	56.4	32.9	176.7
49	V	121.5	8.14	62.4	32.8	176.2
50	Q	124.7	8.53	55.8	29.7	176.1
51	T	116.4	8.28	62.0	69.9	174.5
52	K	124.0	8.43	56.6	33.1	177.0
53	G	110.2	8.46	45.2		174.0
54	K	122.5	8.27	56.3	33.1	176.9
55	R	122.7	8.51	56.4	30.8	176.9
56	G	110.5	8.50	45.2		173.9
57	A	123.9	8.19	52.5	19.5	177.9
58	K	120.5	8.41	56.5	33.1	177.2
59	G	109.9	8.40	45.3		174.2
60	K					
61	Q	121.5	8.46	56.1	29.4	175.8
62	A	124.9	8.33	52.7	19.3	177.7
63	E	120.0	8.36	56.7	30.3	176.6
64	V	120.6	8.09	62.2	32.9	175.9
65	A	127.2	8.37	52.7	19.3	177.5
66	N	117.7	8.40	53.5	38.8	175.2
67	Q	120.3	8.35	56.1	29.5	176.0
68	E	121.8	8.50	56.8	30.3	176.7
69	T	115.8	8.23	61.8	69.9	174.4
70	K	124.1	8.41	56.3	33.1	176.4
71	E	122.1	8.45	56.4	30.5	175.9
72	D	121.7	8.40	54.2	41.2	175.7
73	L	123.7	8.14	53.0	41.9	175.1
74	P					
75	A	124.4	8.41	52.5	19.3	178.1
76	E	120.3	8.47	56.6	30.2	176.4
77	N	119.5	8.51	53.4	39.1	175.8
78	G	109.2	8.41	45.5		174.2
79	E	120.5	8.30	56.6	30.5	176.8
80	T	116.4	8.29	62.1	69.8	174.4

81	K	124.9	8.47	56.1	33.2	176.6
82	T	116.6	8.32	61.9	69.9	174.5
83	E	123.4	8.52	56.4	30.5	176.2
84	E	122.4	8.47	56.4	30.5	176.3
85	S	118.7	8.47	56.5	63.3	179.5
86	P					
87	A	124.7	8.47	52.5	19.3	177.9
88	S	115.2	8.33	58.3	64.0	174.5
89	D	122.5	8.42	54.4	41.1	176.5
90	E	121.2	8.33	56.9	30.1	176.6
91	A	124.5	8.31	53.0	19.2	178.4
92	G	107.8	8.28	45.4		174.3
93	E	120.7	8.28	56.7	30.3	176.8
94	K	122.1	8.37	56.5	33.0	176.7
95	E	121.8	8.37	56.5	30.3	176.1
96	A	125.7	8.33	52.4	19.2	177.5
97	K	121.4	8.33	56.2	33.4	176.5
98	S	118.2	8.46	58.2	64.2	173.4
99	D	127.9	8.06	55.8	42.1	173.8

8.2) Secondary Cβ chemical shift plot for unmodified HMGN1.

Secondary chemical shifts were calculated by subtraction of the respective random coil chemical shift from the observed chemical shift. Random coil chemical shifts were obtained from:

Wishart, D. S.; Bigam, C. G.; Holm, A.; Hodges, R. S.; Sykes, B. D., 1H, 13C and 15N random coil NMR chemical shifts of the common amino acids. I. Investigations of nearest-neighbor effects. *J. Biomol. NMR* **1995,** *5* (1), 67-81.

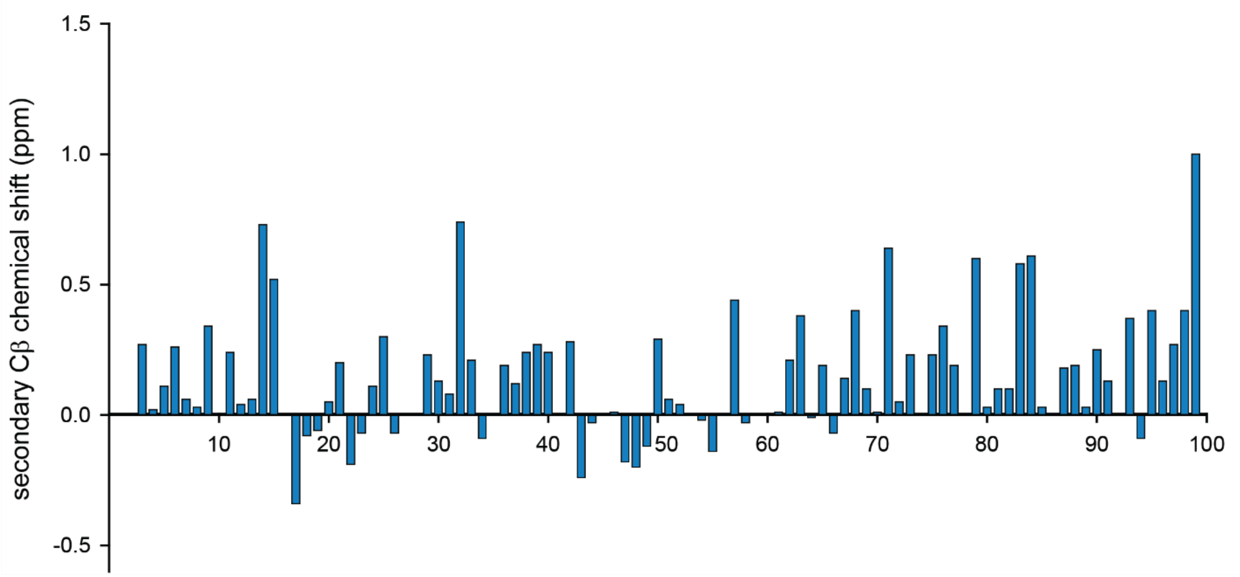

8.3) Secondary C′ chemical shift plot for unmodified HMGN1.

Secondary chemical shifts were calculated by subtraction of the respective random coil chemical shift from the observed chemical shift. The secondary shift for reside D99 (marked with *) is -2.52 ppm.

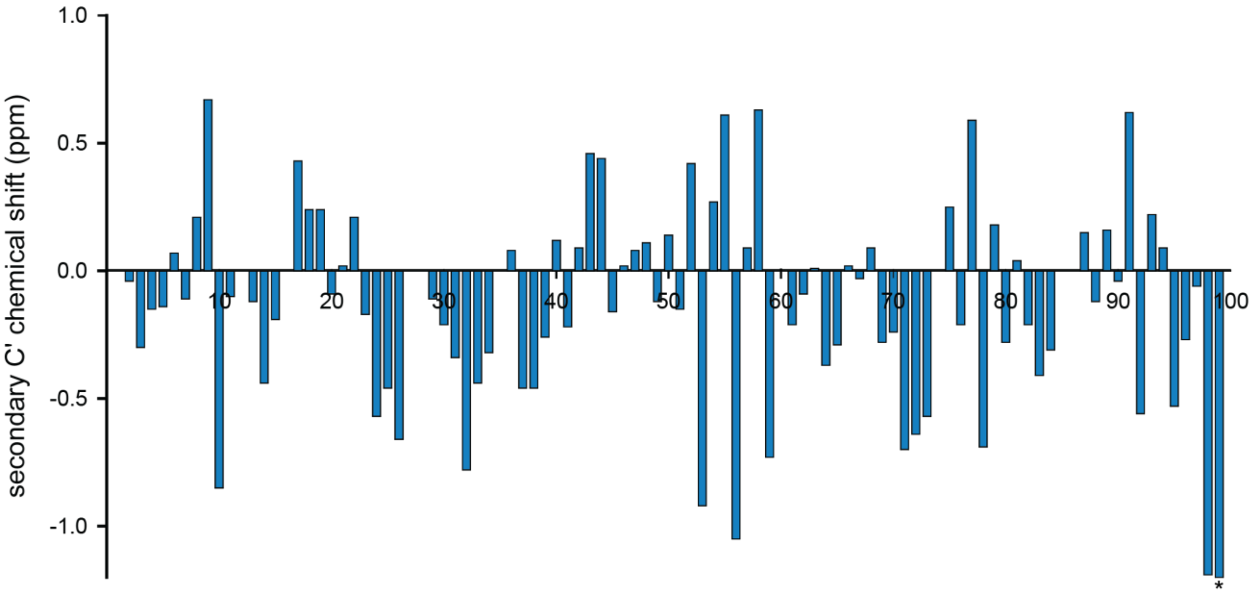

9. Nucleosome and DNA binding assay gels

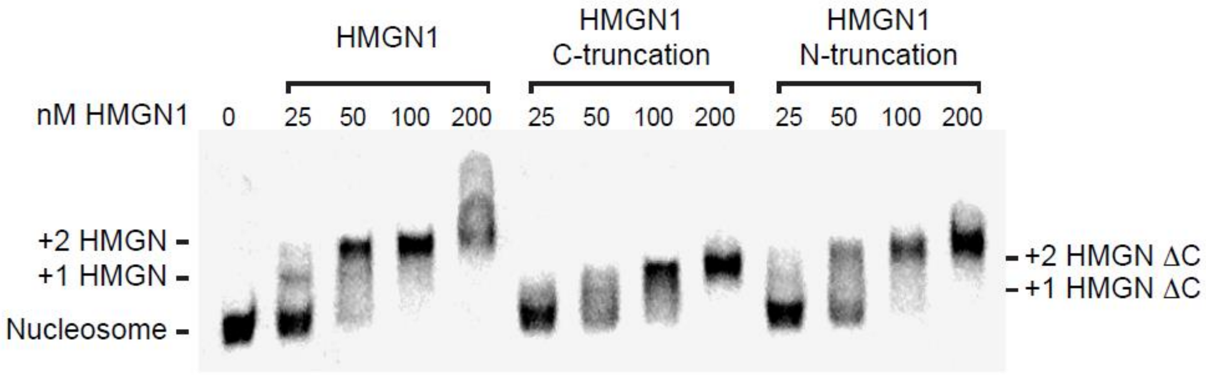

S9.1: Electrophoretic mobility shift assays showing mononucleosome binding of full length HMGN1 and truncated versions used for ligation reactions. Position of nucleosomes bound by one HMGN1 molecule (+1 HMGN), and two HMGN1 molecules (+2 HMGN) are shown in relation to the unbound nucleosome. Binding reactions were separated on 5% TBE-acrylamide gels and then scanned for fluorescence.

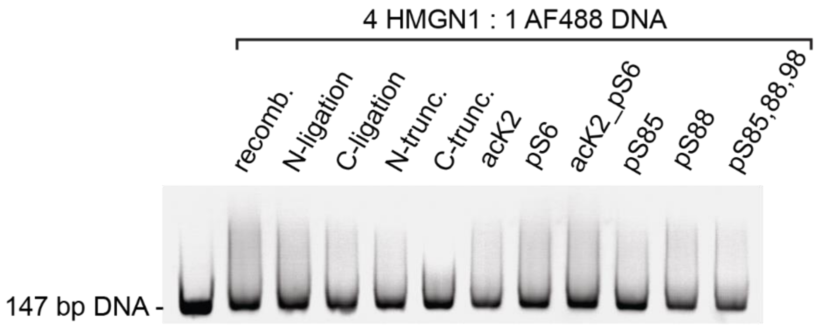

S9.2: Electrophoretic mobility shift assays showing binding of full length, truncated and modified HMGN1 variants to 147 bp AlexaFluor488 labelled DNA containing the 601 nucleosome positioning sequence. Binding reactions were separated on 5% TBE-acrylamide gels and then scanned for fluorescence.

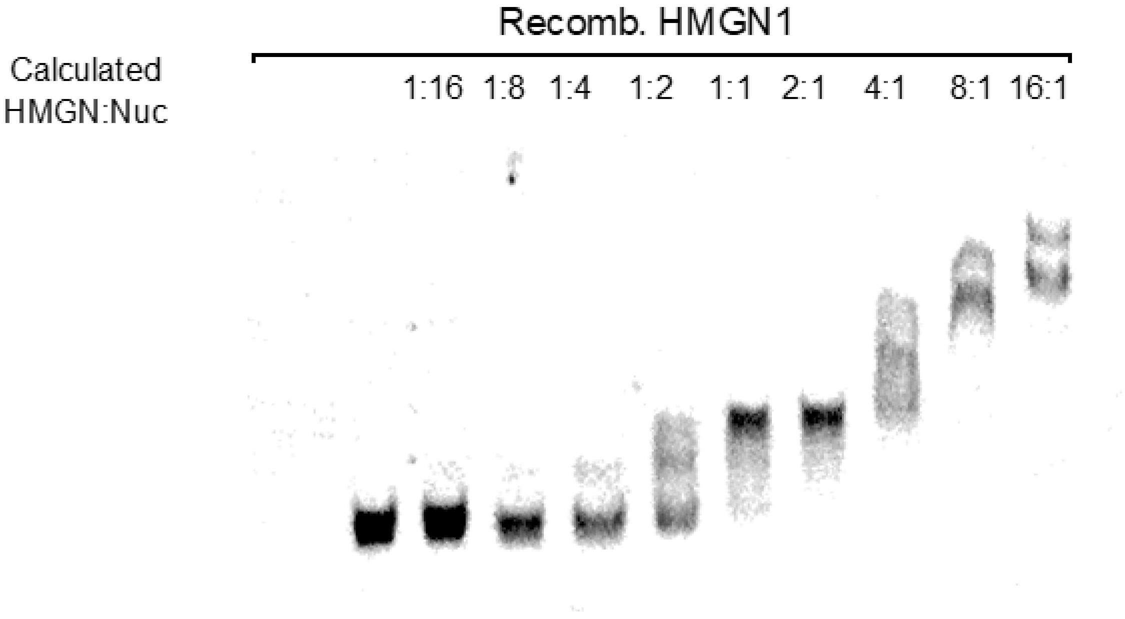

S9.3 Electrophoretic mobility shift assay showing titration of full length unmodified HMGN1 at varying ratios to nucleosome cores (50 nM) assembled on 147 bp AlexaFluor488 labelled DNA containing the 601 nucleosome positioning sequence. Binding reactions were separated on 5% TBE-acrylamide gels and then scanned for fluorescence.

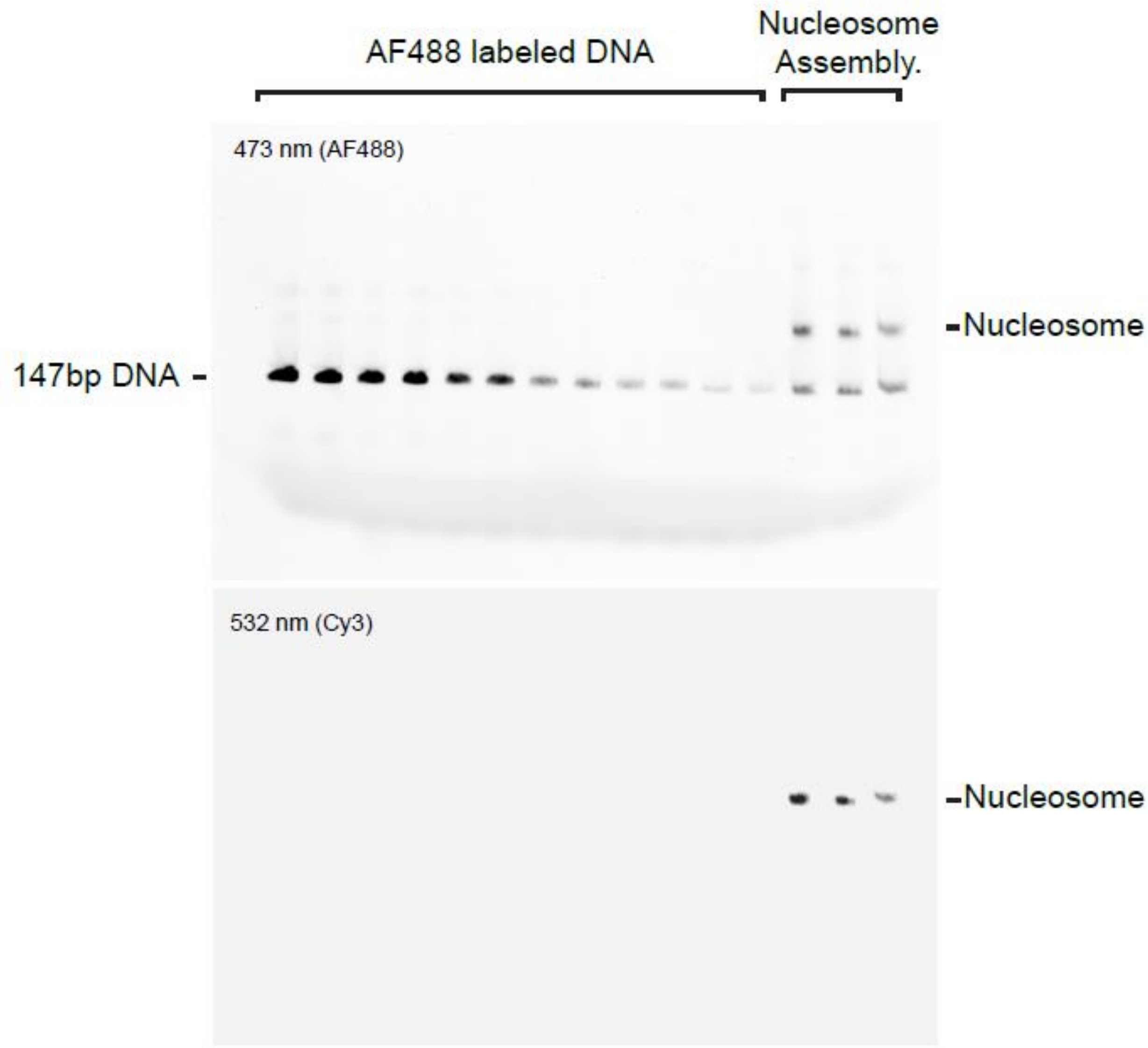

S9.4 Representative native PAGE gel showing a dilution series of 147 bp DNA (left) and reconstituted nucleosomes (right) assembled from AF-488 labelled DNA and three different ratios of Cy3-labelled histone octamers. The nucleosomes showing a single sharp band were used for the electrophoretic gel mobility shift assays.
